# Bio-informatics analysis of a gene co-expression module in adipose tissue containing the diet-responsive gene *Nnat*

**DOI:** 10.1186/1752-0509-4-175

**Published:** 2010-12-27

**Authors:** Xinzhong Li, Peter A Thomason, Dominic J Withers, James Scott

**Affiliations:** 1 National Heart and Lung Institute, Medicine Department, Imperial College London, South Kensington, Exhibition Road, London SW7 2AZ, UK; 2 Beatson Institute for Cancer Research, Garscube Estate, Switchback Road, Glasgow, G61 1BD, UK; 3 Metabolic Signalling Group, Medical Research Council Clinical Sciences Centre, Imperial College London, W12 0NN, UK

## Abstract

**Background:**

Obesity causes insulin resistance in target tissues - skeletal muscle, adipose tissue, liver and the brain. Insulin resistance predisposes to type-2 diabetes (T2D) and cardiovascular disease (CVD). Adipose tissue inflammation is an essential characteristic of obesity and insulin resistance. Neuronatin (*Nnat*) expression has been found to be altered in a number of conditions related to inflammatory or metabolic disturbance, but its physiological roles and regulatory mechanisms in adipose tissue, brain, pancreatic islets and other tissues are not understood.

**Results:**

We identified transcription factor binding sites (TFBS) conserved in the *Nnat *promoter, and transcription factors (TF) abundantly expressed in adipose tissue. These include transcription factors concerned with the control of: adipogenesis (*Pparγ, Klf15, Irf1, Creb1, Egr2, Gata3*); lipogenesis (*Mlxipl, Srebp1c*); inflammation (*Jun, Stat3*); insulin signalling and diabetes susceptibility (*Foxo1, Tcf7l2*). We also identified *NeuroD1 *the only documented TF that controls *Nnat *expression. We identified *KEGG *pathways significantly associated with *Nnat *expression, including positive correlations with inflammation and negative correlations with metabolic pathways (most prominently oxidative phosphorylation, glycolysis and gluconeogenesis, pyruvate metabolism) and protein turnover. 27 genes, including; *Gstt1 *and *Sod3*, concerned with oxidative stress; *Sncg *and *Cxcl9 *concerned with inflammation; *Ebf1, Lgals12 *and *Fzd4 *involved in adipogenesis; whose expression co-varies with *Nnat *were identified, and conserved transcription factor binding sites identified on their promoters. Functional networks relating to each of these genes were identified.

**Conclusions:**

Our analysis shows that *Nnat *is an acute diet-responsive gene in white adipose tissue and hypothalamus; it may play an important role in metabolism, adipogenesis, and resolution of oxidative stress and inflammation in response to dietary excess.

## Background

The WHO estimates there will be 2.3 billion overweight or obese adults world-wide by 2015; including 60% of adults and alarmingly 25% of children in developing countries http://www.who.int. This overweight and obesity epidemic is predicted to affect half the global population by 2025[1,2], largely because of increased urbanization, adaptation of the western diet, and sedentary lifestyle, particularly in China and India.

A serious and nearly ubiquitous complication of obesity is defective insulin signalling in target tissues - skeletal muscle, liver, adipose tissue and brain. Insulin resistance unites obesity with a major disease cluster - diabetes, fatty liver and heart disease - through compensatory hyperinsulinaemia leading to pancreatic *β*-cell failure, and complicating dyslipidaemia and hypertension, major risk factors for CVD. T2D affects 150 million people world-wide, and is estimated to double by 2025[1,2]. CVD is predicted to be the leading cause of death globally by 2020[3,4]. Dementia and cancer are also more common in the obese[4,5]. Therefore, understanding the mechanisms underlying obesity and insulin resistance is of great significance, as the need for new and more effective treatments grows.

Insulin resistance in obesity is causally associated with macrophage influx and low-grade, metabolic inflammation in adipose tissue. Macrophage-driven chronic inflammation in adipose tissue from the obese is considered to be triggered by endoplasmic reticulum (ER) and oxidative stress, together with hypoxia consequent on adiposity expansion and inadequate blood supply. Cellular stress leads to activation of stress kinesis and inflammatory effectors such as *Jnk *and *Nfkb*, with systemic release of cytokine and fatty acid mediators, which inhibit insulin signalling in target tissues[1-7].

While appetite, energy expenditure and metabolism are all critically regulated by hypothalamic brain circuits, white adipose tissue (WAT) is the major storage site for fat in the form of triglycerides, and therefore forms a repository balancing energy intake and expenditure. The overall adipose tissue burden of an individual is a combination of adiposity number (i.e. adipogenesis) and lipid load per cell (i.e. lipogenesis), and both processes may contribute to the adipose tissue mass in obesity.

*Nnat *is a paternally expressed imprinted gene first discovered in the developing brain and it is implicated in antenatal brain development and foetal growth[6-9]. In adult rodents and humans *Nnat *is highly expressed in neurons in many brain regions, including hypothalamic nuclei concerned with appetite regulation, and is concerned in nutrient sensing, and responds to feeding/fasting and leptin[10,11]. *Nnat *is also highly expressed in adult white adipose tissue and aortic endothelial cells, where expression is increased in obese and diabetic rodents[12,13]. *Nnat *is moreover expressed in pancreatic *β*-cell lines, where expression is glucose-dependent, and reduced in diabetic rodents[14-16]. By contrast *Nnat *expression in WAT is substantially reduced in lipodystrophic, in lean *S6kko *mice and in mice fed conjugated linoleic acid, which induces weight loss[16-18]. Recently a consistent association between single nucleotide polymorphisms in the human *Nnat *gene and severe obesity in both children and adults has been demonstrated[10]. This observation suggests that altered *Nnat *function in adipose tissue or the hypothalamic regions regulating energy homeostasis may be involved in the pathogenesis of human obesity.

*Nnat *has three exons, and resides within a large intron of *Blcap*[7,8], tissue specific enhancers lie well upstream of the CpG-rich *Nnat *promoter. Knowledge about the upstream regulation of *Nnat *is confined to the β-cell and control by *NeuroD1*, a transcription factor important for neuronal and endocrine cell differentiation and survival[14].

Knowledge concerning downstream effecter mechanisms through which *Nnat *operates, and its physiological functions are limited. *Nnat *is an amphipathic proteolipid. It has two isoforms, α of 81 and *β *of 54 amino acids, generated by alternative splicing of the middle exon, encoding a membrane-binding domain[13-15,19]. Both isoforms reside in the endoplasmic ER membranes, the *β*-isoform is implicated in ER stress and *β*-cell apoptosis[15], it is considered to regulate ER calcium ATPase through homology to other proteolipid. *Nnat *expression *in vitro *is associated with calcium-induced 3T3-L1 cell adipogenesis; glucose-stimulated, calcium-induced, insulin secretion from *β*-cells; activation of Pi3k, Erk, mTor and calcium signalling in medulloblastoma; *Nfkb-*regulated inflammation in aortic endothelial cells and protection against mitochondrial toxins and ionophores in *PC12 *cells and resolution of injury after ischemic/reperfusion of the kidney [12-14,19,20].

The physiological roles of *Nnat *in the brain, pancreas, adipose and other tissues are not understood. Through genome-wide gene expression analysis we sought to improve our understanding of the mechanisms of *Nnat *regulation and its biological functions. First we carried out an integrated bioinformatics analysis of the conserved *Nnat *promoter in seven species and derived a candidate list of potential *Nnat *transcription factors. Then we interrogated multi-tissue gene expression data to look for primary evidence on the applicability of potential *Nnat *transcriptional regulators. We then used Gene Set Enrichment Analysis[21] to understand which biological functions are most highly correlated with *Nnat *expression. Finally, we generated a mouse protein-protein interaction network in adipose tissue to probe the biological relationships among genes forming a co-expression network with *Nnat*.

## Methods

### Microarray datasets and meta-analysis

In this study, we used four different microarray gene expression datasets from WAT of *C57BL/6J *mice, as described below, to complete a meta-analysis. The first dataset designated BAIR was obtained as part of the Biological Atlas of Insulin Resistance (BAIR, http://www.bair.org.uk) project, a multi-lab collaborative effort to define gene expression changes involved in insulin signalling and resistance. Male mice were weaned at 3 weeks of age and fed a control chow diet containing 5% w/w fat, 19% protein and 3.5% fibre (S&K Universal Ltd, Hull, UK). At 5 weeks of age, a group of mice was transferred to 40% w/w high-fat diet, of which saturated fat is 40%, containing 32% pig fat, 8% casein fat, 19% protein, 21% glucose (Special Diet Services, Witham, UK) *ad libitum*, whereas the control group remained on chow. Mice fat-fed for 2 days, 8 days, 3 weeks and 15 weeks, and with age-matched controls, were killed and epididymal WAT was obtained for microarray gene expression studies, employing *Affymetrix *mouse genome 4302 microarray. From these four time points a total of 40 Affymetrix gene expression raw *CEL *files were produced and *RMA *normalized by using the *Affy *package http://www.bioconductor.org; present/absent call information was also calculated based on *Affymetrix MAS5 *algorithm. This dataset has been deposited in EBI ArrayExpress Database with accession number E-BAIR-12 http://www.ebi.ac.uk/microarray-as/ae/.

The second dataset was obtained from the Gene Expression Omnibus (GEO, http://www.ncbi.nlm.nih.gov/geo/, accession ID: GSE4671), which contains 28 Affymetrix mouse genome 4302 chips. Nine-week old male mice in this experiment were fed with either a control chow diet or a chow diet that also contained 0.5% conjugated linoleic acid (*CLA*). At each of seven time points (day 1, 2, 3, 4, 7, 10, 17), retroperitoneal WAT was obtained from two control mice and two test mice. Microarray data were processed using GCRMA algorithm.

The third dataset was also acquired from GEO with accession number GSE8831, in which 20 female and 15 male C57BL/6J mice, fed by *ad libitum*, varied in body weight and insulin sensitivity were studied. Fasting blood glucose and serum insulin concentrations were measured 2-4 days prior to collection of adipose tissue. *RNA *from perigonadal adipose tissue was extracted and processed for analysis on *Affymetrix Mu74Av2 *microarrays. *RMA *normalization was applied to the 35 raw CEL files, and *MAS5 *present/absent call information was calculated as well.

The fourth dataset consisted of 13 *Affymetrix MG-U74av2 *chips for epididymal WAT extracted from six-week old C57BL/6J male mice from the Diabetes Genome Anatomy Project DGAP http://www.diabetesgenome.org. There were four high-fat feeding mice (derives 55% calories from fat, 21% calories from protein, 24% calories from carbohydrates), nine low-fat feeding control mice (derives 14% calories from fat, 25% calories from protein and 61% calories from carbohydrates). Gene expression data was *RMA *normalized with present/absent call information.

In order to identify the gene co-vary with *Nnat *in WAT, we calculated Pearson correlation coefficients between the expression of *Nnat *and all other genes in each of the above four datasets separately, and chose genes showing the highest correlation with *Nnat *expression. Subsequently, rather than merely combining the p-values of the Pearson correlation coefficients, we used weighted unbiased Pearson statistical values, i.e. we normalized the statistical values to approximate a normal distribution with unit variance and zero mean. Some necessary filters were designed for quality control: (a) probesets with expression value lower than 100 or (b) absolute Pearson value less than 0.2 or (c) present/absent call less than 30% were excluded. Each gene was represented by the probesets which had the largest absolute Pearson correlation in each dataset in order to remove multiple annotated probesets for the same gene. In this way we obtained 2841, 3061, 2148 and 1201 unique genes within the four datasets respectively, giving 6120 unique genes in total.

The following formula was applied to calculate the combined *Z-score*

$$Z_{score}=\sum_{i=1}^{4}\left(\sqrt{\omega_{i}}\times T_{i}\right)/\sqrt{\sum_{i=1}^{4}\omega_{i}}$$

*ω_i _*denotes the number of samples in each dataset (i.e. 40, 28, 35, 13); *T_i _*indicates the normalized Pearson association test statistic based on a *t *distribution. A two-tailed *P*-value can be derived by the normal distribution for each gene from this combined *Z_score_*. *Bonfferroni *corrected *FDR *< 0.05 was applied, yielding the top 34 genes which are most strongly co-expressed with *Nnat*. For comparison we also applied *Fisher's *Z-transform to combine the transformed Pearson correlation coefficients in the meta-analysis, we got similar results. For the multi-species analysis, seven genes, *Acp5, Tcta, Znrf2, Serpina3c, S3-12, Sgce *and *Lrg1*, which are not conserved in rat, were excluded.

### Promoter analysis

To delineate the *Nnat *promoter, we identified the conserved non-coding region of the *Nnat *locus in seven mammalian genera (*Pan, Bos, Canis, Mus, Sus, Rattus *and *human*). This corresponds to 1200 *bp *upstream and 800 *bp *downstream of the most utilized transcription start points (TSP) of *Nnat*, identified through the presence of CAGED transcripts in mice and human. For example, mouse *Nnat *has six transcripts, with two promoters having respectively 36 and 28 CAGE tags, while human *NNAT *has four transcripts with one promoter having 29 CAGE tags. For these analyses we used the Genome VISTA genome alignment tool http://www-gsd.lbl.gov/vista/ in conjunction with *Genomatix Gene2promoter *tool (*Genomatix *Software Inc., Ann Arbor, MI, USA). The *Genomatix MatInspector *programme with default parameter settings was used to detect common Transcription Factor Binding Sites (TFBS) between the seven promoter sequences of the seven genera. The occurrence of the TFBSs within all vertebrate genes was used as a control in *MatInspector*. Significant common TFBSs among the seven species were defined by having a p-value < 0.05 and presence in six or more species, compared with the control sets in the *Genomatix *database. We further explored the TF genes in each TFBSs family, examining expression data to determine whether they were expressed in human or mouse adipose, hypothalamus and pancreas islet (using *BioGPS *database http://biogps.gnf.org and our own BAIR data as reference sources). Promoter analysis was also applied to these 27 fully conserved *Nnat *co-expression genes (plus *Nnat*) all together, focusing on human, mouse and rat only. Again, their expression status in adipose, hypothalamus and pancreatic islets were included as well.

### Gene set enrichment analysis

In order to inspect which biological pathways are significantly correlated with *Nnat *expression in adipose tissue, we employed Gene Set Enrichment Analysis, using the GSEA package http://www.broadinstitute.org/gsea/ for each of the above four datasets separately. We chose Pearson correlation and permuted 1000 times to detect statistical significance. Expression data were pre-filtered by using the present/absent call and background correction, similarly to the pre-process in meta-analysis above. In the GSEA database there were 200 *KEGG *pathway sets and 639 canonical pathway sets. We first focused on those approximately 100 *KEGG *gene sets which have more than 15 genes and fewer than 500 genes; the results returned were significant in the above four datasets as well as an additional dataset which included 45 sleep mice model with hypothalamus tissue (GEO: GSE6514). We then worked on those canonical pathway sets which passed the same filter only on BAIR fat-fed mice and human fat dataset about 20 lean and 19 obese person[22].

### Protein-protein interaction network analysis

By using BioNetBuilder, a cytoscape plugin package http://err.bio.nyu.edu/cytoscape/bionetbuilder/ with supported databases DIP, BIND, Prolinks, KEGG, HPRD, The BioGrid and GO, we thus generated a mouse protein-protein interaction network which had 11777 unique nodes (genes) and 107813 unique edges (links). For the 27 fully conserved *Nnat *co-expression genes, we searched each of their direct correlation genes (first neighbours) in the PPI network, again filtered by expression value > 200 in adipose tissue by *BioGPS*.

## Results

### *Nnat *promoter analysis

We sought to investigate mechanisms of transcriptional regulation of *Nnat *and of co-varying genes in adipose tissue. The neuronatin gene is located on human chromosome 20 and mouse chromosome 2. To define the *Nnat *promoter we described the conserved non-coding region in seven mammalian species, including mouse and human, corresponding to 1200 bp upstream and 800 bp downstream of the most utilized transcription start points (TSP) of *Nnat *identified through the presence of CAGED transcripts in mice and humans. We used *Genomatix *software to determine the presence of binding sites for TF families in the conserved *Nnat *promoter, across seven mammalian species. We then filtered these potential regulators based on their expression in mouse WAT, and compared their expression also in hypothalamus and pancreatic *β*-cells in which *Nnat *is known to be expressed. Table 1 shows the gene expression behaviour for members of TFBS families scoring as significant for presence in the *Nnat *promoter (p-value < 0.05), and present in at least six of the *Nnat *promoter sequences; expression values were filtered on the basis of *BioGPS *expression bigger than 200, which therefore focuses attention on the most abundantly expressed TFs.

**Table 1:** Expression of conserved TFBS in Nnat promoter

TFs	TFBSs	p-value	WAT	hypo	panc	TFs	TFBSs	p-value	WAT	hypo	panc
Pparγ	V$PPAR	0	11959	253	357	Hsf2	V$HEAT	0.0202	271	543	279
Zfp161	V$ZF5F	2E-05	1309	1537	523	Tef	V$PARF	0.0209	4845	5297	846
Rreb1	V$RREB	0.0005	3530	1344	117	Myb	V$MYBL	0.0217	730	51	81
Tead4	V$TEAF	0.0005	271	239	367	Meox2	V$HBOX	0.0221	555	37	47
Hes2	V$HESF	0.0016	468	304	498	Stat3	V$STAT	0.0236	3754	1438	617
Gtf2i	V$DICE	0.002	5309	7006	398	Myt1l	V$MYT1	0.0247	327	1039	395
Zfp384	V$CIZF	0.0023	1966	989	1073	Neurod1	V$NEUR	0.0262	119	323	224
Myog	V$MYOD	0.0024	523	524	638	Neurod2	V$NEUR	0.0262	209	216	237
Glis2	V$GLIF	0.0026	308	365	390	Hand1	V$HAND	0.0283	557	519	662
Mef2d	V$MEF2	0.0027	2451	4184	668	Bcl6	V$BCL6	0.0301	4569	1672	82
Tcf7l2	V$LEFF	0.0033	4252	1922	329	Gata3	V$GATA	0.0323	432	54	72
Ctcf	V$CTCF	0.004	2655	1586	890	Pbx2	V$PBXC	0.0345	524	456	253
Srf	V$SRFF	0.0041	340	275	212	Hoxd9	V$ABDB	0.0364	230	139	190
Zfp219	V$ZBPF	0.0042	1282	1712	270	Hoxc8	V$HOXF	0.0371	1804	203	364
Hif1a	V$HIFF	0.0042	542	564	194	Evi1	V$EVI1	0.0412	274	240	335
Mlxipl	V$EBOX	0.006	304	27	32	Creb1	V$CREB	0.0415	2387	1157	532
Nfat5	V$NFAT	0.0069	1154	842	203	Jun	V$CREB	0.0415	2503	1955	1735
Egr2	V$EGRF	0.0078	408	141	289	Klf15	V$KLFS	0.0419	7360	2193	2438
Tfdp1	V$E2FF	0.0125	1993	1575	77	Klf4	V$KLFS	0.0419	4878	593	348
Pou6f1	V$BRN5	0.0146	632	973	446	Klf6	V$KLFS	0.0419	3219	583	895
Sp1	V$SP1F	0.0147	322	200	306	Foxo1	V$FKHD	0.0424	400	129	106
Dmrt2	V$DMRT	0.0157	275	28	57	Sox4	V$SORY	0.043	4829	1217	74
Nfyc	V$CAAT	0.0161	2207	914	247	Pou2f1	V$OCT1	0.0443	225	229	238
Irf1	V$IRFF	0.0166	5486	259	196	Elf1	V$ETSF	0.0464	2419	78	134
Ahr	V$AHRR	0.0171	483	135	62	Nr2f2	V$NR2F	0.0472	6090	8397	259
Nfia	V$NF1F	0.0171	3505	2335	505	Rxrb	V$RXRF	0.0479	1715	1309	1944
Lhx4	V$LHXF	0.0177	269	242	225	Msx1	V$HOMF	0.0479	392	360	435

Conserved TFBS found in ***Nnat ***promoter region across seven mammalian species and their gene expression in three tissues of mice.

The TFBS for the *V$NEUR *family (including *NeuroD1 *and *NeuroD2*) was found to be highly significant in six out of seven promoter sequences, but it was not found in the rat promoter sequences in our defined region. However, *V$NEUR *does exist farther upstream from the rat *Nnat *transcription start site (within 2.5kb). *NeuroD1*, the only confirmed transcriptional regulator of *Nnat *expression[14] is not one of the most highly expressed *TFs *in adipose, though its expression value is similar across all three tissues shown in Table 1, it is highly expressed in cerebellum.

The binding sites for the key adipose tissue regulator *Pparγ *are the most significant in the seven *Nnat *promoter sequences. Of the TFs with conserved binding sites it is also by far the most highly expressed in WAT, and has a crucial role in adipocyte metabolism. Other important TFs with conserved binding sites in the *Nnat *promoter and which are highly expressed in WAT include several genes which have crucial roles in the control of: adipogenesis (*Klf15, Irf1, Creb1, Egr2, Gata3*); lipogenesis (*Mlxipl*, *Srebp1c*); inflammation (*Jun, Stat3*); insulin signalling and diabetes susceptibility (*Foxo1, Tcf7l2*). Interestingly, the *Rreb1 *has a binding site in all seven *Nna*t promoters, and is expressed at high levels in adipose and hypothalamus. *Rreb1 *can enhance the transcription-activating function of *NeuroD1*, a property that requires both the physical interaction of *Rreb1 *with *NeuroD1*, and binding *of Rreb1 *to *DNA*[23]. Notable by their absence are binding sites for the C/EBP family of TFs, clearly suggesting that this family (several members of which are required for adipocyte differentiation) are not concerned with *Nnat *regulation, and may be down-stream of *Nnat*.

### Identification of biological pathways associated with *Nnat *expression

In order to determine which biological pathways are significantly correlated with *Nnat *expression in WAT and hypothalamus, we employed Gene Set Enrichment Analysis. Initially we focused on *KEGG *pathways to investigate those processes that are most highly associated with *Nnat *expression in WAT and hypothalamus. In WAT, there are five *KEGG *pathways significantly correlated with *Nnat *expression (FDR < 0.05) in at least two of the four mouse WAT datasets (Table 2), these five pathways are all significant in BAIR fat-fed data, and include oxidative phosphorylation, ribosome, proteasome, cholera infection and neuroactive ligand receptor interaction, these last two indicating perturbation of ion transport and neuronal function. Furthermore, oxidative phosphorylation, ribosome and cholera infection are significant in the mouse hypothalamus dataset as well. Additional file 1, **Figure S1 **illustrates the enrichment plots and positively and negatively correlated genes in these three common pathways. Additional file 1, **Table S1 **lists the core enriched genes that correlate with *Nnat *in these three common pathways in both WAT and hypothalamus. GSEA on the canonical pathways (Additional file 1, **Table S2**) reveals that *Nnat *expression in mice on a high-fat diet and obese models displayed highly positive correlations with inflammatory pathways (complement and coagulation, cytokine and integrin signalling), and strongly negative correlations with metabolic pathways (most prominently oxidative phosphorylation, pyruvate metabolism, glycolysis and gluconeogenesis) and protein turnover (including proteasome, ribosome and mRNA processing). In human adipose tissue *NNAT *mRNA expression is decreased (Additional file 1, **Table S3**). In consequence *NNAT *expression displayed strong positive correlation with genes involved in protein turnover and fatty acid metabolism/degradation, and negatively correlated with genes involved in adhesion, complement and coagulation and cytokine. Additional file 1, **Table S4 **lists the common pathways correlated with *Nnat *expression between BAIR fat-fed mice and human obese WAT, and these are concerned with cytokine signalling and cell-cell interaction. Additional file 2, **Table S5 **contains the complete list of genes associated with *Nnat *in each *KEGG *pathway in mouse models.

**Table 2:** Pathways associated with Nnat

*KEGG *Pathway	Size	p-value	FDR	Correlation	Tissue
OXIDATIVE_PHOSPHORYLATION	92	< 0.001	< 0.001	Negative	WAT
RIBOSOME	52	< 0.001	< 0.001	Negative	WAT
PROTEASOME	22	< 0.001	< 0.001	Negative	WAT
CHOLERA_INFECTION	33	< 0.001	< 0.01	Negative	WAT
NEUROACTIVE_LIGAND_RECEPTOR_INTERACTION	36	< 0.001	< 0.01	Positive	WAT
OXIDATIVE_PHOSPHORYLATION	93	< 0.001	0.011	Negative	Hypo
RIBOSOME	52	< 0.001	0.006	Negative	Hypo
CHOLERA_INFECTION	32	< 0.001	0.031	Positive	Hypo
ALANINE_AND_ASPARTATE_METABOLISM	25	< 0.002	0.029	Positive	Hypo
ARGININE_AND_PROLINE_METABOLISM	18	< 0.001	0.026	Positive	Hypo
TRYPTOPHAN_METABOLISM	31	< 0.001	0.025	Positive	Hypo
SELENOAMINO_ACID_METABOLISM	21	0.004	0.029	Positive	Hypo
THYROID_CANCER	21	0.003	0.025	Positive	Hypo
BUTANOATE_METABOLISM	29	0.004	0.036	Positive	Hypo
GALACTOSE_METABOLISM	16	0.006	0.04	Positive	Hypo
GLYCOLYSIS_AND_GLUCONEOGENESIS	30	0.004	0.05	Positive	Hypo
PATHOGENIC_ESCHERICHIA_COLI_INFECTION_EHEC	24	0.008	0.049	Positive	Hypo

Significant pathways associated with *Nna*t analyzed by GSEA.

### Identification of genes that co-vary with Nnat

The genes whose expression co-varies with *Nnat *are likely to share regulatory mechanisms and, perhaps, biological functions. We therefore identified genes whose expression is highly correlated with *Nnat *in a meta-analysis of WAT gene expression data. Table 3 shows the correlation of gene expression behaviour with that of *Nnat*, in four microarray datasets; entries with a null value indicate that this gene is missing in the specific dataset; their fold change information in BAIR fat-fed mice and human with obesity are shown in Table 4. Additional file 1, **Figure S2 **gives the hierarchical clustering for these 28 genes in BAIR fat-fed dataset, here genes with expression most closely associated with *Nnat *include *Aqp1, Sncg, Sulf2 *and *Cxcl9*. Additional file 1, **Figure S3 **illustrates the expressions of the top four most correlated genes (*Gstt1, Ccdc80, Hfe Sod3*) with *Nnat *in BAIR and GSE6571 datasets. Among these genes is only one transcription factor, *Ebf1 *which has previously been suggested to have a regulatory role in adipogenesis[24]. Another of the *Nnat*-correlated genes, *Lgals12*, is a member of the galectin family of beta-galactoside-binding proteins, and is a major regulator of adipose tissue development[25].

**Table 3:** Co-expression genes with Nnat in WAT

Genes	GSE8831	GSE4671	BAIR	DGAP	meta.z	p-value	FDR
GSTT1	0.33	0.92	0.87	0.71	6.16	7.50E-10	4.59E-06
CCDC80	0.36	0.93	0.86		5.82	5.85E-09	3.58E-05
HFE	0.71	0.93	0.71		5.81	6.37E-09	3.90E-05
SOD3	0.64	0.84	0.79	0.51	5.55	2.93E-08	0.0002
NPDC1	0.67	0.92	0.62	0.7	5.44	5.47E-08	0.0003
AQP1	0.68	0.71	0.8	0.47	5.37	7.73E-08	0.0005
EBF1	0.62	0.84	0.81		5.3	1.15E-07	0.0007
SNCG	0.81	0.58	0.72		5.28	1.30E-07	0.0008
LGALS12		0.95	0.83		5.15	2.57E-07	0.0016
SLC6A13	0.73	0.8	0.7	0.4	5.1	3.43E-07	0.0021
HEBP1	0.73	0.84	0.69		5.09	3.53E-07	0.0022
NPR3	0.66	0.71	0.77	0.54	5.07	4.04E-07	0.0025
RAD50	0.63	0.35	0.85		5.06	4.16E-07	0.0025
H6PD	0.54	0.85	0.77	0.53	5.04	4.57E-07	0.0028
HTRA3	0.48	0.95	0.67		5.01	5.76E-07	0.0035
ART3	0.63	0.81	0.77		4.95	7.28E-07	0.0045
CAV1	0.67	0.73	0.78		4.95	7.46E-07	0.0046
AOC3	0.27	0.95	0.76	0.25	4.94	7.73E-07	0.0047
ANXA6	0.65	0.73	0.73	0.57	4.84	1.29E-06	0.0079
CXCL9	0.33	0.53	0.88		4.71	2.51E-06	0.0154
JUP	0.55	0.79	0.62	0.89	4.7	2.60E-06	0.0159
BLCAP	0.5	0.9	0.61	0.76	4.65	3.30E-06	0.0202
FZD4	0.49	0.73	0.79	0.5	4.63	3.62E-06	0.0221
SULF2		0.88	0.86		4.63	3.68E-06	0.0226
CD151		0.83	0.87	0.46	4.62	3.79E-06	0.0232
MAP1LC3A	0.68	0.78	0.59	0.67	4.56	5.05E-06	0.0309
EPN2	0.54	0.91	0.53	0.71	4.48	7.42E-06	0.0454

High significant co-expression genes with *Nnat *in WAT based on gene expression meta-analysis. This table shows the Pearson correlation coefficient value of each gene in each of the four microarray datasets, blank value means that the relevant gene is not expressed (not passed the QC or even not included into the microarray chip). Top combined meta-zscore, meta-pvalue and FDR test are shown by FDR < 0.05.

**Table 4:** The fold change of co-expression genes

Genes	BAIR.2d	BAIR.8d	BAIR.3w	BAIR.15w	Human
NNAT	7.82	3.54	1.02	1.19	0.72
GSTT1	2.5	1.43	1.1	0.89	0.86
CCDC80	2.65	2.2	0.69	1.05	2.57
HFE	1.59	1.16	0.77	1.34	0.87
SOD3	1.99	1.64	0.87	1.45	0.89
NPDC1	1.28	1.14	0.92	0.96	1.88
AQP1	1.64	1.32	1.27	1.01	1.52
EBF1	2.07	1.54	0.89	1.28	0.68
SNCG	6.16	8.06	1.53	1.53	1.26
LGALS12	3.12	1.47	0.89	0.93	1.03
SLC6A13	2.09	1.74	0.54	1.89	0.77
HEBP1	1.35	1.18	0.77	1	1.08
NPR3	5.28	3.14	0.7	2.87	1.69
RAD50	2.02	1.67	0.85	1.24	0.71
H6PD	1.82	1.38	1.05	0.95	1.16
HTRA3	1.65	1.23	0.8	1.27	1.12
ART3	2.03	1.41	0.92	0.88	1.33
CAV1	1.48	1.18	0.88	1.14	0.93
AOC3	1.91	1.11	0.75	1.37	0.83
ANXA6	1.64	1.59	0.97	1.4	0.85
CXCL9	3.48	2.3	1.26	1.25	1.22
JUP	1.54	1.07	0.89	1	1.06
BLCAP	1.58	1.11	1.03	1.1	0.92
FZD4	2.24	1.42	0.88	1.9	1.11
SULF2	1.59	1.31	1	1.11	2.08
CD151	1.54	1.23	0.95	1.18	1.1
MAP1LC3A	1.32	0.95	1.03	1.32	0.93
EPN2	1.14	1.03	1.18	1.17	1.24

This table shows the fold change (FC) of those co-expression genes of Nnat in adipose tissue in fat-fed mice during development of diet-induced obesity, and in human obese adipose tissue.

In order to investigate whether these 27 genes share conserved TFBS with *Nnat *as identified in Table 1, we used *Genomatix *tools to analyze the promoters of all 28 genes in human, mouse and rat separately. We found that nine TFBS families in Table 1, *V$CREB, V$EVI1, V$GATA, V$KLFS, V$NR2F, V$RXRF, V$SORY, V$STAT, V$HAND*, which are conserved in the promoter region of *Nnat *from seven species, are significant TFBS of all these 28 genes in human, mouse and rat, indicating these TFBSs are likely to be important in WAT. We also found that *V$RREB *and *V$TEAF *are highly significant TFBS*s *(p-value < 3.31e-10, p-value < 6.22e-8, p-value < 5.58e-7) in most of the 28 genes, while *V$PPAR *is highly significant (p-value < 5.7e-4, p-value < 4.14e-6, p-value < 5.7e-4) half in of the 28 genes (Table 5).

**Table 5:** Common TFBS among Nnat and its co-vary genes

TFBS	hs_pval	mm_pval	rat_pval	#hs	#mm	#rat
V$PPAR	5.70E-04	4.14E-06	5.70E-04	12	15	12
V$ZF5F	1.62E-06	2.34E-04	0.0033	18	15	13
V$RREB	3.31E-10	6.22E-08	5.58E-07	26	24	23
V$TEAF	6.22E-08	6.22E-08	5.34E-09	24	24	25
V$STAT	9.89E-09	1.29E-07	8.77E-06	26	25	23
V$HESF	2.35E-09	4.30E-08	2.83E-05	27	26	23
V$MYOD	1.29E-08	1.69E-05	9.92E-05	27	24	23
V$GLIF	5.36E-09	3.90E-06	3.00E-07	28	26	27
V$MEF2	0.0025	2.52E-05	0.0025	21	24	21
V$LEFF	0.0042	5.25E-05	2.80E-04	21	24	23
V$CTCF	9.37E-05	1.43E-05	1.59E-06	24	25	26
V$SRFF	1.50E-05	0.002	1.67E-06	25	22	26
V$ZBPF	1.23E-06	2.30E-08	1.23E-06	27	28	27
V$HIFF	2.45E-07	8.69E-12	2.45E-07	22	26	22
V$EBOX	8.80E-06	6.89E-05	1.34E-08	26	25	28
V$NFAT	5.79E-05	5.79E-05	0.0025	27	27	25
V$EGRF	1.84E-04	2.35E-06	1.84E-04	25	27	25
V$SP1F	8.94E-06	8.94E-06	8.94E-06	27	27	27
V$CAAT	2.19E-06	2.19E-06	1.00E-04	28	28	27
V$CART	0.0029	0.0029	0.0029	25	25	25
V$IRFF	6.53E-04	0.0034	6.53E-04	26	25	26
V$NF1F	4.37E-06	5.72E-08	4.37E-06	23	25	23
V$AHRR	6.01E-07	6.07E-08	1.42E-04	24	25	21
V$HEAT	0.0015	2.07E-04	2.07E-04	26	27	27
V$MYBL	0.0021	3.12E-04	7.05E-06	26	27	28
V$STAT	1.38E-05	1.38E-05	1.38E-05	28	28	28
V$NEUR	1.64E-04	2.64E-05	3.09E-06	24	25	26
V$HAND	2.30E-04	2.30E-04	2.30E-04	28	28	28
V$BCL6	5.24E-06	0.0027	3.39E-05	24	20	23
V$GATA	6.49E-05	6.49E-05	6.49E-05	28	28	28
V$EVI1	3.23E-04	3.23E-04	3.23E-04	28	28	28
V$CREB	3.64E-04	3.64E-04	3.64E-04	28	28	28
V$KLFS	6.56E-04	6.56E-04	6.56E-04	28	28	28

The common TFBS among the 28 (*Nnat *and its co-vary) genes in three species respectively. The last three columns give the number of promoter sequences among the 28 which share the same TFBS in their promoter sequences

**Figure 1:**
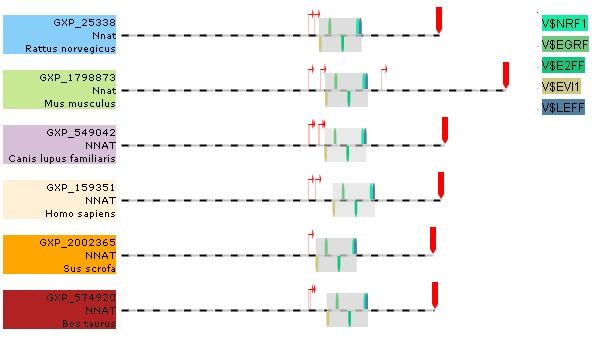
**Transcription factor module in six *Nnat *promoter sequences**. There are 20 significant five-element modules found in the six promoter sequences of *Nnat *for six species (Human, Mus, Can, Sus, Bos, Rat), sequence in 1200 bp upstream and 800 bp downstream, here shows TFBSs for V$EVI1 - V$EGRF - V$E2FF - V$NRF1 - V$LEFF, on the *Nnat *promoter closely and together in the similar region of the six species separately just upstream of the major TSP.

We used the *Genomatix *Promoter Module Library and were able to identify highly conserved models of five transcription factor elements highly conserved across the *Nnat *promoter. Among these promoter sequences, we got 20 significant models which consist of elements from five transcription factor family. Figure 1 shows TFBPs for V$EVI1 - V$EGRF - V$E2FF - V$NRF1 - V$LEFF, on the *Nnat *promoter close together in a similar region for six species separately just upstream of the major TSP. V$EVI1 - V$EGRF - V$E2FF and -V$LEFF are in Table 1 and TF members of these families are expressed in WAT, hypothalamus and pancreas. V$EVI1 - V$EGRF and - V$LEFF in Table 5 indicate conservation in the promoters of genes that co-vary with *Nnat*. There are important transcription factors in these families, for example *Egr2 *and *Tcf7l2*, which are implicated in stress responses and T2D; *E2f *which is concerned with adipogenesis and *Evi1*, which activates *Stat3*, a transcription factor concerned with leptin signalling in the hypothalamus.

### Functional networks formed by genes that co-vary with Nnat

**Figure 2:**
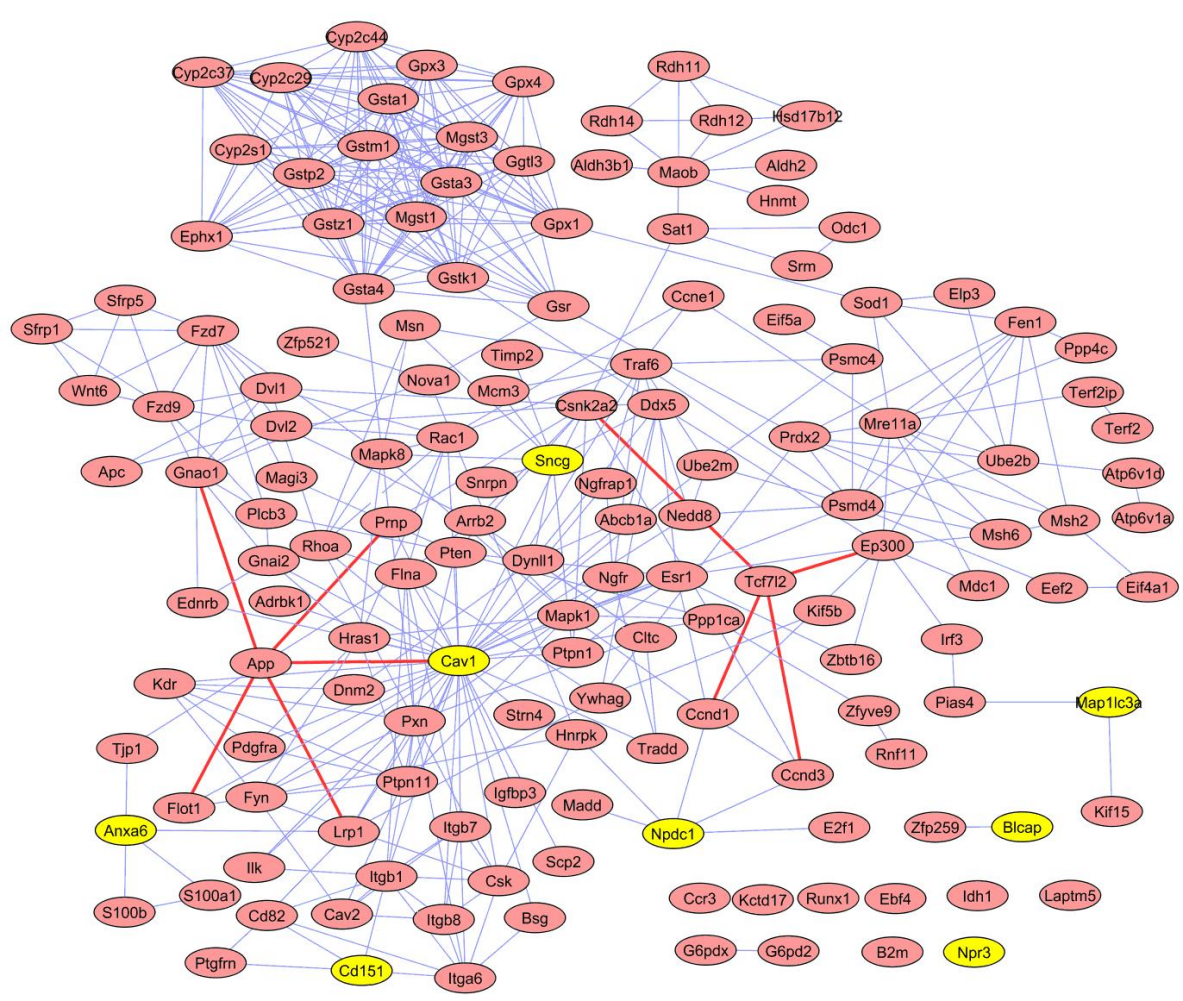
**Mouse *Nnat *co-expression gene tissue-specific PPI network**. All ***Nnat ***co-expression genes in Table 3 and their first neighbours in mouse PPI network showing expressed in WAT, hypothalamus and pancreas, generated a tissue-specific network with 151 nodes and 447 links. The eight genes in yellow are ***Nnat ***co-varying genes in Table 3. Two obesity relative genes ***App ***and ***Tcf7l2 ***and their direct associated genes linked by red edges.

In the mouse PPI network, we sought *Nnat*, and the 27 genes that co-vary with it, and their respective first neighbours. 21 of the 27 *Nnat *co-expression genes can be mapped to this PPI network, which contains 375 first neighbours, leading to a sub-network with a total of 396 nodes and 3804 edges. Among these genes, 191 (48%) are expressed in hypothalamus (including 11 of the 21 *Nnat *co-varying genes) and 275 (69%) are expressed in WAT (including all 21 of the *Nnat *co-expression genes), 190 (48%) are expressed in pancreas (including 8 of the 21 *Nnat *co-varying genes, all expressed in hypothalamus as well). The functional groups formed by the PPI involving the 21 *Nnat *co-varying genes (examples given in parentheses with sentinel gene emboldened) correspond to the following major biological functions: (1) cellular stress, inflammation, adipocyte metabolism, metabolic syndrome and type 2 diabetes (*Sncg, Erk1/2, Jnk, Hnf4a *and *Fabp4*); (2) detoxification of aldehydes, retinol and products of oxygen free radical metabolism (*Aoc3, Aldh1a3/9a*1, *Rdh11/12/13*); (3) local natriuretic peptide system and growth (*Npr3, Nppa*); (4) glutathione and xenobiotic metabolism (*Gstt1/3, Cyp1a1/1b1*); (5) iron metabolism (*Hfe *and *Tfrc*); (6) *Wnt *signaling (*Fzd6, Sfrp1 *and *Wtn11*); (7) adipogenesis (*Ebf1, Ebf4, Crebbp, Ep300 *); (8) protein turnover (*Rad50, Eif4a1, Psmc4 *and *Ube2b*); (9) integrins, neurodegeneration, and metabolic, inflammatory and growth-control signalling (*Cav1, Ngfr, Tnfrsf1b, Hras1, Pten, App, Hnf4a *and *Esr*). Figure 2 shows a sub-network with genes expressed in WAT, hypothalamus and pancreatic β-cells, which has 151 nodes and 447 links. Eight genes that co-vary with *Nnat *in the three tissues are shown in yellow, and comprise: *Anxa6, Cav1, Cd151, Gstt1, Map1lc3a, Npdc1, Npr3, and Sncg*. *Nnat *does not appear since it is not included in the mouse PPI database.

## Discussion

This study explored the behaviour of the diet-responsive gene *Nnat *in multiple gene expression datasets from mouse adipose tissue, and subsequently analysed *in silico *the promoter of *Nnat *in seven mammalian species. Rosen and MacDougald[24] have pointed out that at least 100 TFs are expressed in adipocyte, and our analyses here have sought to focus attention on those which could play a part in the regulation of expression of *Nnat *and its co-varying genes. Based on our study we find 50 TFBSs conserved across seven species on the *Nnat *promoter, and expression of the TFs corresponding to these in adipose tissue, many of which have known roles in adipocyte processes including, adipogenesis (*Klf15, Irf1, Creb1, Egr2, Gata3*); lipogenesis (*Mlxipl, Srebp1c*); inflammation (*Jun, Stat3*); insulin signalling and diabetes susceptibility (*Foxo1, Tcf7l2*). These TFs are also well expressed in the hypothalamus and pancreas.

*NeuroD1 *is the only confirmed transcription factor for *Nnat*, whose expression regulates in the developing pancreas[14]. Binding sites for the TF family *V$NEUR *(which includes *NeuroD1 *and *NeuroD2*) are predicted in six of the seven mammalian *Nnat *proximal promoter regions examined here (all except rat), and are also present further upstream in the rat *Nnat *promoter region. Members of this family are expressed at reasonable levels in all three mouse tissues analysed here, so it remains possible that the *V$NEUR *family participates in *Nnat *regulation in several tissues. *NeuroD1 *is considered to be a weak activator of transcription, but is known to cooperate with other transcription factors, including *Rreb1 *and *Sp1*[23,26], in the stimulation of gene expression. Binding sites for both of these families are conserved in the *Nnat *promoter across mammalian species, as well as in the promoters for the *Nnat *co-varying genes analysed above. Indeed this property requires both the physical interaction of *Rreb1 *with *NeuroD1*, and binding *of Rreb1 *to DNA. Direct evidence for such a mechanism of regulation requires experimental validation. The expression of *Nnat *remains strong in the developing pituitary of *NeuroD1 *knockout mice[14], so it is clear that different TFs (or combinations thereof) are likely to be of varying importance in regulating *Nnat *expression in different tissues.

*Pparγ *is both necessary and sufficient for adipocyte differentiation of fibroblast/pre-adipocyte cell lines[24] and the presence, in all the *Nnat *promoters analysed here, of *Pparγ *binding sites is consistent with it playing a role in regulation of *Nnat *expression in WAT. *Nnat *expression is strongly reduced in WAT of a mouse model in which a dominant negative version of *Pparγ *is expressed[17]. Activation of *Pparγ *with *Pparγ *agonists improves insulin signalling and decreases inflammation. This is consistent with the view that *Nnat *protects against toxins and is concerned with resolution of tissue injury. Indeed our unpublished results in 3T3L1 cells, where we have over expressed *Nnat *indicate an anti-inflammatory function. However there is currently no evidence on whether *Pparγ *plays a direct role in the regulation of *Nnat*. Likewise, in mice expressing an inactive mutant version of *Srebp1c*, *Nnat *expression in WAT is severely reduced as well[17]. In both of these mouse models complex changes in WAT gene expression make it difficult to draw conclusions as to the regulation of particular genes by specific TFs. *Pparγ *is known to operate in a cellular network with other TFs of the *EBF, C/EBP*, *KLF *and *IRF *families in regulating adipogenesis[24,27-29]. Our bioinformatical analysis suggests that *Pparγ *could be a candidate for *Nnat *regulation, meanwhile the absence of *V$SREB *binding sites in the *Nnat *promoter does not support a direct role for *Srebp1c*.

The pattern of gene expression observed in the mouse WAT upon high fat feeding in experiments analysed here suggests a critical in oxidative stress; indeed sentinel indicators of this *Gstt1, Ccdc80, Hfe *and *Sod3 *all concerned with cellular management of free radicals are the genes most highly correlated with *Nnat *in the meta-analysis (Additional file 1, **Figure S3**). Genes concerned with adipogenesis (*Ebf1, Fzd4 *and *Lgals12*) and inflammation (*Sncg, Cxcl9 *and *Aoc3*) are also highly correlated in mouse WAT upon high fat feeding (Additional file 1, **Figure S2**). This is consistent with data indicating that *Nnat *expression favours adipogenesis and inflammation. Of the aforementioned adipogenic TFs, only *Ebf1 *expression correlates well with *Nnat*, but the conserved *Nnat *promoter does not possess *Ebf1 *binding sites (TF family *V$NOLF*). Thus it is unlikely that *Ebf1 *regulates *Nnat *expression; instead they may both be targets of the same TFs. By treating the *Nnat *co-varying genes as a set, and carrying out promoter analysis on this set, we discovered that many wide-acting TFs have binding sites in all of these genes. This does not itself indicate which, if any, of these TFs may regulate the *Nnat *co-varying genes, but does draw attention to the families *V$HIFF *(example: *HIF1a*) *V$RREB *(example: *Rreb1*) and *V$TEAF *(example: *Tead4*) as being the most highly over-represented TFBS in this group of genes.

Our gene set enrichment analysis showed that expression of *Nnat *is strongly negatively correlated with energy metabolism (most notably oxidative phosphorylation) and positively correlated with inflammation. Repression of oxidative phosphorylation gene expression is one of the major changes seen in human diabetes[21], and a metabolic/inflammatory network is thought to underlie metabolic syndrome in susceptible mice[30]. Although in the human data *NNAT *expression is not correlated with oxidative phosphorylation in the GSEA in a more general analysis of gene expression in this human data the oxidative phosphorylation gene set is enriched. We would expect that major changes in gene set regulation require the participation of wide-acting TFs and/or extensive networks of TFs and their cofactors.

We found 27 genes that show strong co-variation with *Nnat*. 21 of these can be mapped to the PPI network, and eight of these (*Anxa6, Cav1, Cd151, Gstt1, Map1lc3a, Npdc1, Npr3, and Sncg*) are expressed in adipose tissue, pancreas and hypothalamus. *App *and *Tcf7l2*, genes implicated in metabolic inflammation, Alzheimer's disease and diabetes are shown linked to their respective first neighbours (Figure 2). *Sncg *along with *Nnat *is a leptin responsive gene[11].

## Conclusions

In conclusion we identify numerous TFBSs, conserved among mammals, on the *Nnat *promoter, and expression of TFs corresponding to these in adipose tissue, hypothalamus and pancreas. Many of these have known roles in adipocyte, as well as pancreatic and hypothalamic processes. We observe clustering of TFBSs for *Egr1 *and *Tcf7l2*, which are implicated in stress responses and T2D; *E2f *which is concerned with adipogenesis and *Evi1*, a developmental gene highly expressed in pancreatic islets. GSEA analysis suggests the roles for *Nnat *in oxidative stress, resolution of inflammation, energy metabolism, mainly oxidative phosphorylation, and protein turnover. Genes that co-vary with *Nnat*, such as *Sncg, Gstt1, Hfe *and *Ebf1*, share some of these TFBSs and form functional networks. The recent demonstration that SNPs in *Nnat *are found in obese humans suggest that altered *Nnat *function in adipose tissue or the hypothalamus may be involved in the pathogenesis of obesity via such mechanisms.

## Authors' contributions

XL and JS conceived and designed this study, XL and PT performed the data analysis, XL, PT, DJW and JS wrote the manuscript. All authors read and approved the final manuscript.

## Supplementary Material

Additional file 1 **This PDF file includes supplementary figures and supplementary table 1-4**. Click here for file

Additional file 2 SupplementaryTable5 Click here for file

## References

[B1] Zimmet P Alberti KG Shaw J Global and societal implications of the diabetes epidemic Nature 2001 414 6865 782 787 10.1038/414782a 11742409

[B2] Saltiel AR New perspectives into the molecular pathogenesis and treatment of type 2 diabetes Cell 2001 104 4 517 529 10.1016/S0092-8674(01)00239-2 11239409

[B3] Breslow JL Cardiovascular disease burden increases, NIH funding decreases Nat Med 1997 3 6 600 601 10.1038/nm0697-600 9176478

[B4] Yach D Stuckler D Brownell KD Epidemiologic and economic consequences of the global epidemics of obesity and diabetes Nat Med 2006 12 1 62 66 10.1038/nm0106-62 16397571

[B5] de la Monte SM Wands JR Alzheimer's disease is type 3 diabetes-evidence reviewed J Diabetes Sci Technol 2008 2 6 1101 1113 19885299 10.1177/193229680800200619 PMC2769828

[B6] Evans HK Weidman JR Cowley DO Jirtle RL Comparative phylogenetic analysis of blcap/nnat reveals eutherian-specific imprinted gene Mol Biol Evol 2005 22 8 1740 1748 10.1093/molbev/msi165 15901842

[B7] Evans HK Wylie AA Murphy SK Jirtle RL The neuronatin gene resides in a "micro-imprinted" domain on human chromosome 20q11.2 Genomics 2001 77 1-2 99 104 10.1006/geno.2001.6612 11543638

[B8] John RM Aparicio SA Ainscough JF Arney KL Khosla S Hawker K Hilton KJ Barton SC Surani MA Imprinted expression of neuronatin from modified BAC transgenes reveals regulation by distinct and distant enhancers Dev Biol 2001 236 2 387 399 10.1006/dbio.2001.0327 11476579

[B9] Sing A Pannell D Karaiskakis A Sturgeon K Djabali M Ellis J Lipshitz HD Cordes SP A vertebrate Polycomb response element governs segmentation of the posterior hindbrain Cell 2009 138 5 885 897 10.1016/j.cell.2009.08.020 19737517

[B10] Vrang N Meyre D Froguel P Jelsing J Tang-Christensen M Vatin V Mikkelsen JD Thirstrup K Larsen LK Cullberg KB The imprinted gene neuronatin is regulated by metabolic status and associated with obesity Obesity (Silver Spring) 2010 18 7 1289 1296 10.1038/oby.2009.361 19851307 PMC2921166

[B11] Tung YC Ma M Piper S Coll A O'Rahilly S Yeo GS Novel leptin-regulated genes revealed by transcriptional profiling of the hypothalamic paraventricular nucleus J Neurosci 2008 28 47 12419 12426 10.1523/JNEUROSCI.3412-08.2008 19020034 PMC2650686

[B12] Mzhavia N Yu S Ikeda S Chu TT Goldberg I Dansky HM Neuronatin: a new inflammation gene expressed on the aortic endothelium of diabetic mice Diabetes 2008 57 10 2774 2783 10.2337/db07-1746 18591389 PMC2551689

[B13] Suh YH Kim WH Moon C Hong YH Eun SY Lim JH Choi JS Song J Jung MH Ectopic expression of Neuronatin potentiates adipogenesis through enhanced phosphorylation of cAMP-response element-binding protein in 3T3-L1 cells Biochem Biophys Res Commun 2005 337 2 481 489 10.1016/j.bbrc.2005.09.078 16223607

[B14] Chu K Tsai MJ Neuronatin, a downstream target of BETA2/NeuroD1 in the pancreas, is involved in glucose-mediated insulin secretion Diabetes 2005 54 4 1064 1073 10.2337/diabetes.54.4.1064 15793245 PMC1197706

[B15] Joe MK Lee HJ Suh YH Han KL Lim JH Song J Seong JK Jung MH Crucial roles of neuronatin in insulin secretion and high glucose-induced apoptosis in pancreatic beta-cells Cell Signal 2008 20 5 907 915 10.1016/j.cellsig.2008.01.005 18289831

[B16] Selman C Tullet JM Wieser D Irvine E Lingard SJ Choudhury AI Claret M Al-Qassab H Carmignac D Ramadani F Ribosomal protein S6 kinase 1 signaling regulates mammalian life span Science 2009 326 5949 140 144 10.1126/science.1177221 19797661 PMC4954603

[B17] Kim S Huang LW Snow KJ Ablamunits V Hasham MG Young TH Paulk AC Richardson JE Affourtit JP Shalom-Barak T A mouse model of conditional lipodystrophy Proc Natl Acad Sci USA 2007 104 42 16627 16632 10.1073/pnas.0707797104 17921248 PMC2034232

[B18] LaRosa PC Miner J Xia Y Zhou Y Kachman S Fromm ME Trans-10, cis-12 conjugated linoleic acid causes inflammation and delipidation of white adipose tissue in mice: a microarray and histological analysis Physiol Genomics 2006 27 3 282 294 10.1152/physiolgenomics.00076.2006 16868072

[B19] Siu IM Bai R Gallia GL Edwards JB Tyler BM Eberhart CG Riggins GJ Coexpression of neuronatin splice forms promotes medulloblastoma growth Neuro Oncol 2008 10 5 716 724 10.1215/15228517-2008-038 18701710 PMC2666248

[B20] Dekel B Metsuyanim S Schmidt-Ott KM Fridman E Jacob-Hirsch J Simon A Pinthus J Mor Y Barasch J Amariglio N Multiple imprinted and stemness genes provide a link between normal and tumor progenitor cells of the developing human kidney Cancer Res 2006 66 12 6040 6049 10.1158/0008-5472.CAN-05-4528 16778176

[B21] Mootha VK Lindgren CM Eriksson KF Subramanian A Sihag S Lehar J Puigserver P Carlsson E Ridderstrale M Laurila E PGC-1alpha-responsive genes involved in oxidative phosphorylation are coordinately downregulated in human diabetes Nat Genet 2003 34 3 267 273 10.1038/ng1180 12808457

[B22] Nair S Lee YH Rousseau E Cam M Tataranni PA Baier LJ Bogardus C Permana PA Increased expression of inflammation-related genes in cultured preadipocytes/stromal vascular cells from obese compared with non-obese Pima Indians Diabetologia 2005 48 9 1784 1788 10.1007/s00125-005-1868-2 16034612 PMC1409821

[B23] Ray SK Nishitani J Petry MW Fessing MY Leiter AB Novel transcriptional potentiation of BETA2/NeuroD on the secretin gene promoter by the DNA-binding protein Finb/RREB-1 Mol Cell Biol 2003 23 1 259 271 10.1128/MCB.23.1.259-271.2003 12482979 PMC140679

[B24] Rosen ED MacDougald OA Adipocyte differentiation from the inside out Nat Rev Mol Cell Biol 2006 7 12 885 896 10.1038/nrm2066 17139329

[B25] Yang RY Hsu DK Yu L Chen HY Liu FT Galectin-12 is required for adipogenic signaling and adipocyte differentiation J Biol Chem 2004 279 28 29761 29766 10.1074/jbc.M401303200 15131127

[B26] Ray SK Leiter AB The basic helix-loop-helix transcription factor NeuroD1 facilitates interaction of Sp1 with the secretin gene enhancer Mol Cell Biol 2007 27 22 7839 7847 10.1128/MCB.00438-07 17875929 PMC2169158

[B27] Eguchi J Yan QW Schones DE Kamal M Hsu CH Zhang MQ Crawford GE Rosen ED Interferon regulatory factors are transcriptional regulators of adipogenesis Cell Metab 2008 7 1 86 94 10.1016/j.cmet.2007.11.002 18177728 PMC2278019

[B28] Birsoy K Chen Z Friedman J Transcriptional regulation of adipogenesis by KLF4 Cell Metab 2008 7 4 339 347 10.1016/j.cmet.2008.02.001 18396140 PMC2430156

[B29] Shao D Lazar MA Peroxisome proliferator activated receptor gamma, CCAAT/enhancer-binding protein alpha, and cell cycle status regulate the commitment to adipocyte differentiation J Biol Chem 1997 272 34 21473 21478 10.1074/jbc.272.34.21473 9261165

[B30] Chen Y Zhu J Lum PY Yang X Pinto S MacNeil DJ Zhang C Lamb J Edwards S Sieberts SK Variations in DNA elucidate molecular networks that cause disease Nature 2008 452 7186 429 435 10.1038/nature06757 18344982 PMC2841398

