# Supplementary material for: Bio-informatics analysis of a gene co-expression module in adipose tissue containing the diet-responsive gene Nnat
Source: BMC Syst Biol. 2010 Dec 27;4:175. doi: 10.1186/1752-0509-4-175 (PMC3022651; doi:10.1186/1752-0509-4-175)
Supplement: Additional file 1 — This PDF file includes supplementary figures and supplementary table 1-4. [file 1752-0509-4-175-S1.PDF]

Supplemental Material

1. **Supplementary Figure 1**. GSEA for BAIR fat-fed mice on WAT and GSE6514 sleeping mouse model on hypothalamus tissue. Here figure a, c, e, g, i relate to WAT and b., d., f, h, j to hypothalamus. Genes in both oxidative phosphorylation and ribosome pathways are involved in energy and protein metabolism respectively. The cholera infection gene set (concerned with fluid and electrolyte transport) has negative correlation with WAT and positive with hypothalamic gene expression.

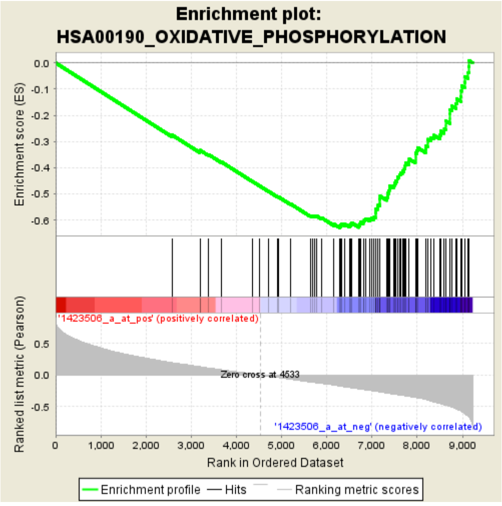

a.

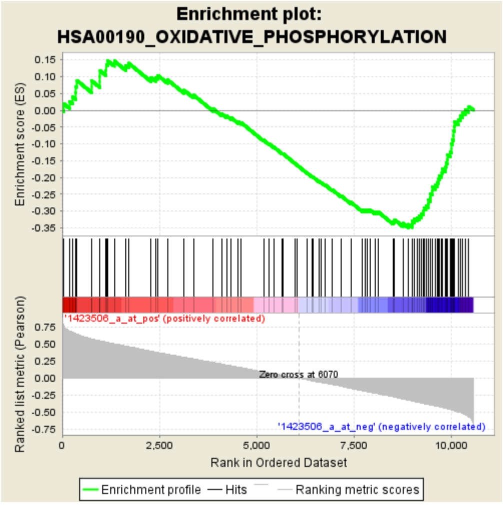

b.

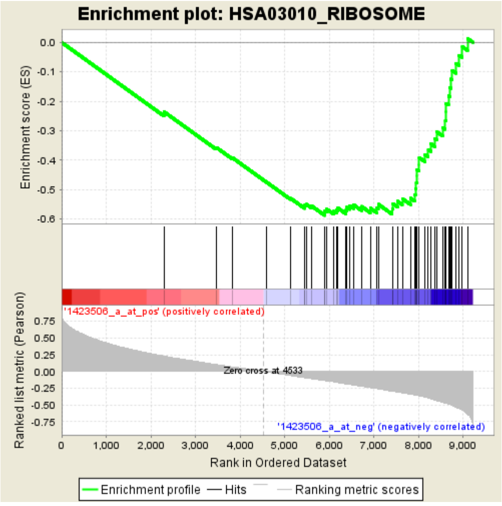

c.

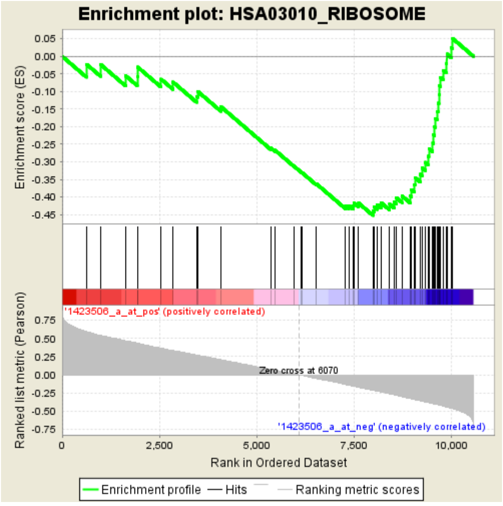

d.

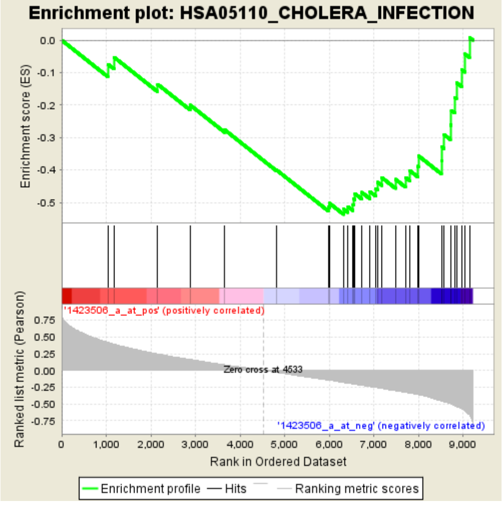
Enrichment plot: HSA05110_CHOLERA_INFECTION
Enrichment score (ES)
Ranked list metric (Pearson)
'1423506_a_at_pos' (positively correlated)
Zero cross at 4533
'1423506_a_at_neg' (negatively correlated)
Rank in Ordered Dataset
Enrichment profile
Hits
Ranking metric scores

e.

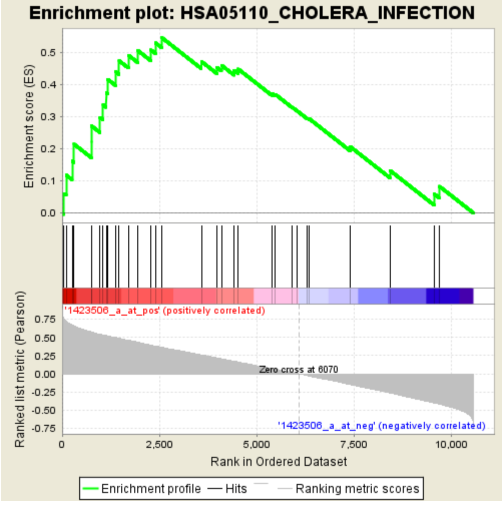
Enrichment plot: HSA05110_CHOLERA_INFECTION
Enrichment score (ES)
Ranked list metric (Pearson)
'1423506_a_at_pos' (positively correlated)
Zero cross at 6070
'1423506_a_at_neg' (negatively correlated)
Rank in Ordered Dataset
Enrichment profile
Hits
Ranking metric scores

f.

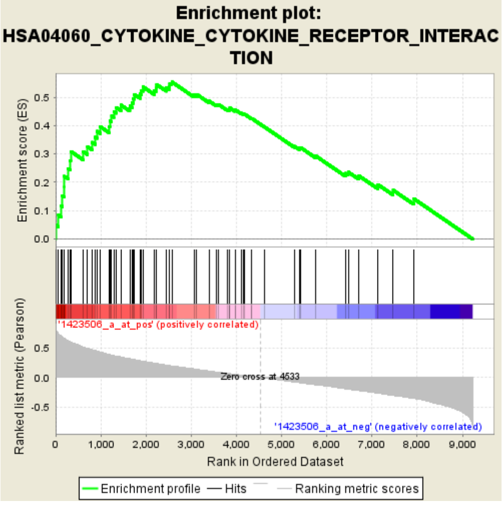
Enrichment plot: HSA04060_CYTOKINE_CYTOKINE_RECEPTOR_INTERACTION
Enrichment score (ES)
Ranked list metric (Pearson)
'1423506_a_at_pos' (positively correlated)
Zero cross at 4533
'1423506_a_at_neg' (negatively correlated)
Rank in Ordered Dataset
Enrichment profile
Hits
Ranking metric scores

g.

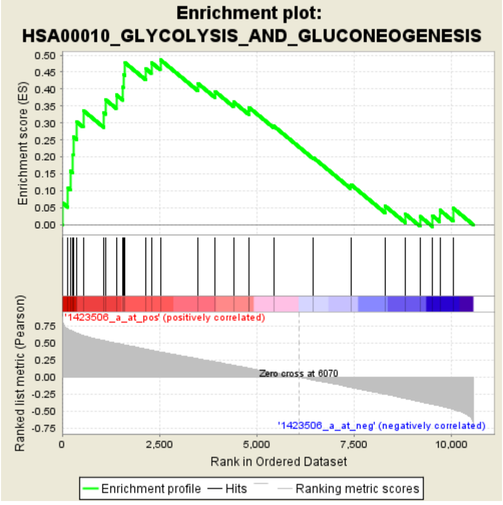
Enrichment plot: HSA00010_GLYCOLYSIS_AND_GLUCONEOGENESIS
Enrichment score (ES)
Ranked list metric (Pearson)
'1423506_a_at_pos' (positively correlated)
Zero cross at 6070
'1423506_a_at_neg' (negatively correlated)
Rank in Ordered Dataset
Enrichment profile
Hits
Ranking metric scores

h.

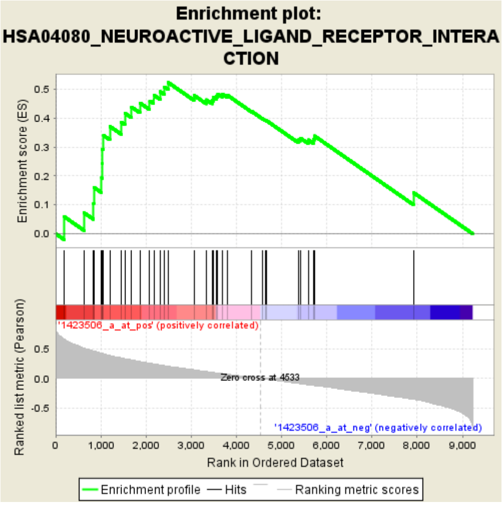
Enrichment plot: HSA04080_NEUROACTIVE_LIGAND_RECEPTOR_INTERACTION
Enrichment score (ES)
Ranked list metric (Pearson)
'1423506_a_at_pos' (positively correlated)
Zero cross at 4533
'1423506_a_at_neg' (negatively correlated)
Rank in Ordered Dataset
Enrichment profile
Hits
Ranking metric scores

i.

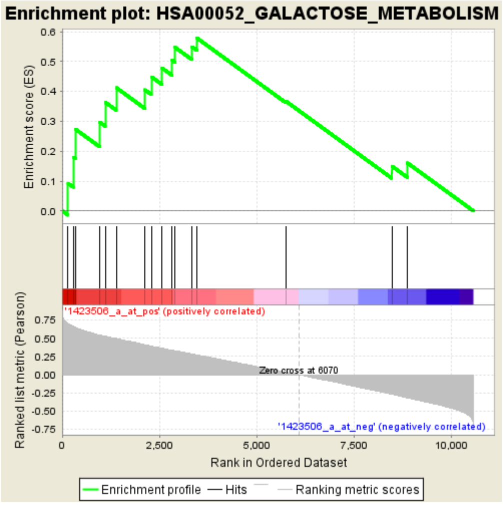
Enrichment plot: HSA00052_GALACTOSE_METABOLISM
Enrichment score (ES)
Ranked list metric (Pearson)
'1423506_a_at_pos' (positively correlated)
Zero cross at 6070
'1423506_a_at_neg' (negatively correlated)
Rank in Ordered Dataset
Enrichment profile
Hits
Ranking metric scores

j.

2. **Supplementary Figure 2.** Hierarchical clustering of fold changes of *Nnat* and its co-variant genes in BAIR fat-fed mice at different time points. Here, colour in red denotes up-regulated and green denotes down-regulated.

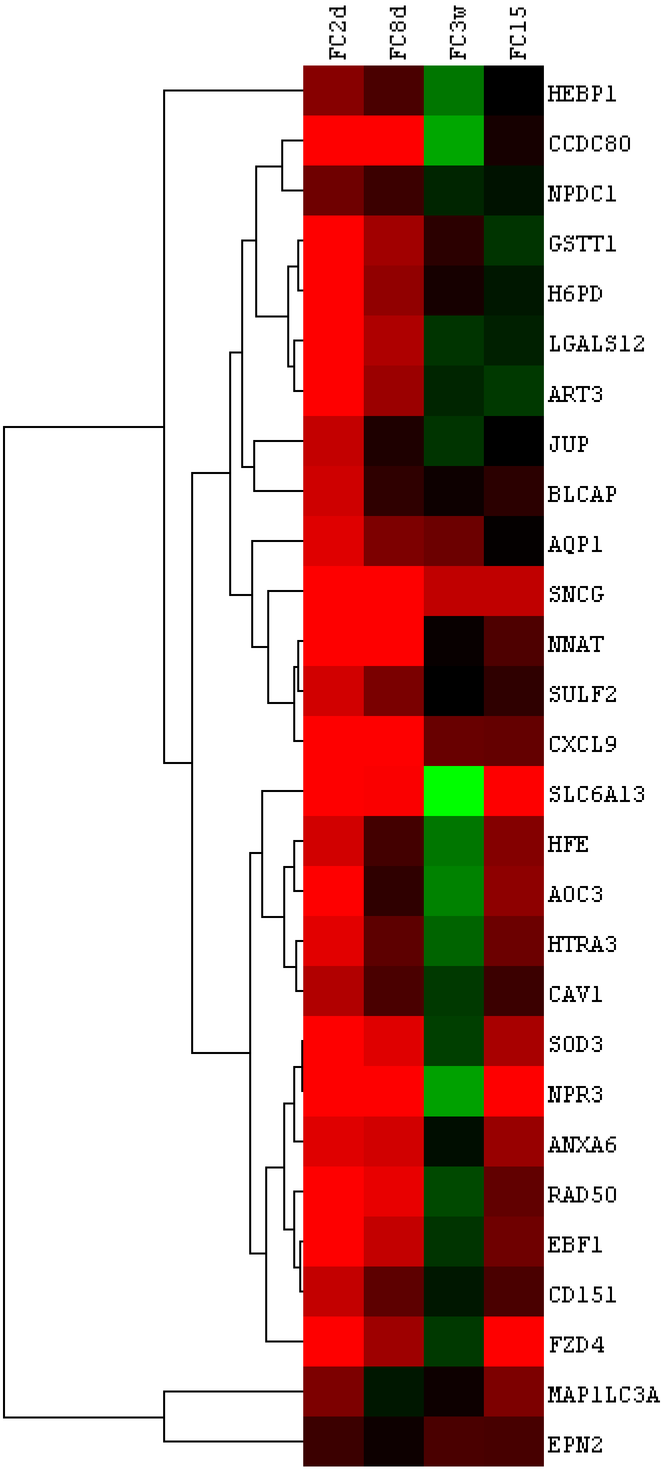

3. **Supplementary Figure 3**. The top four most strongly co-variant genes with *Nnat* in BAIR fat-fed and GSE4651 mouse WAT.

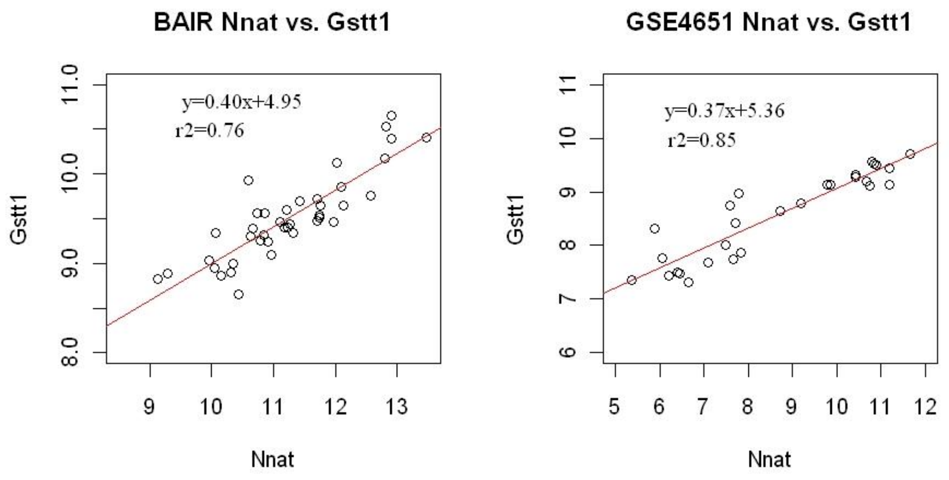

a. Expression of Nnat and Gstt1

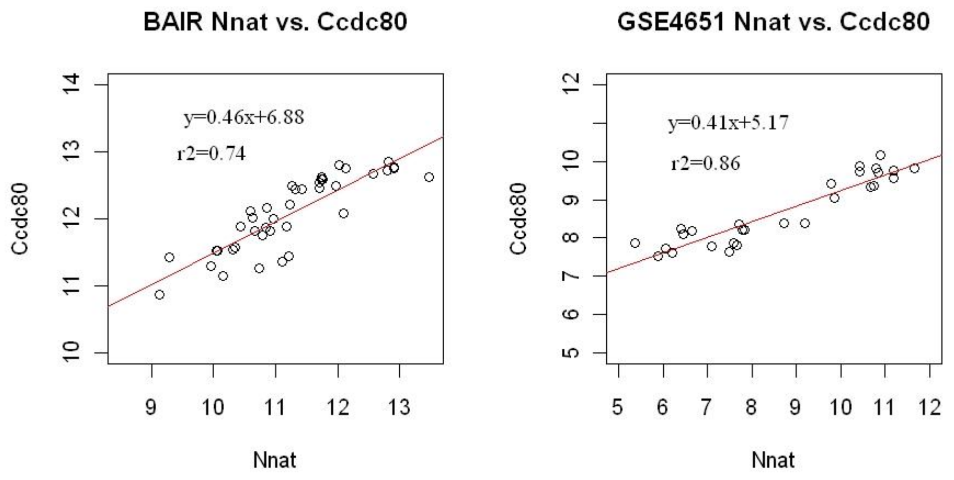

b. Expression of Nnat and Ccdc80

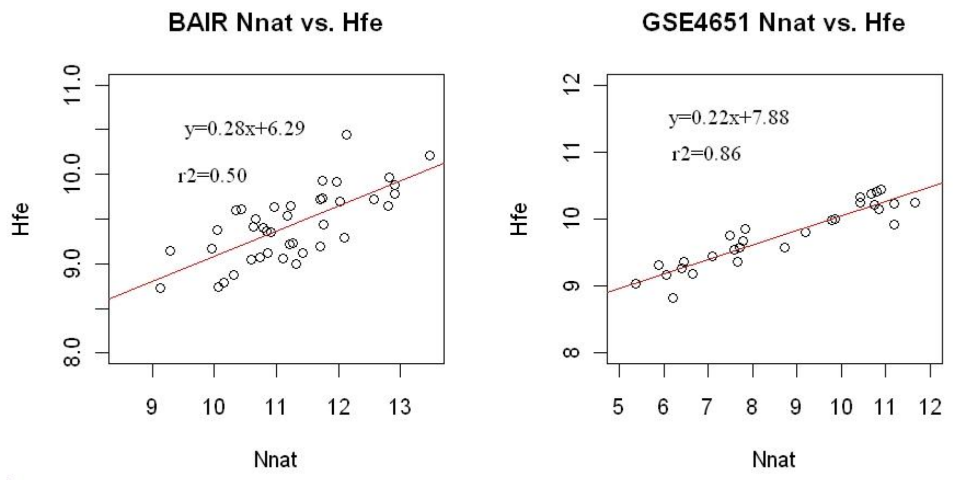

c. Expression of Nnat and Hfe

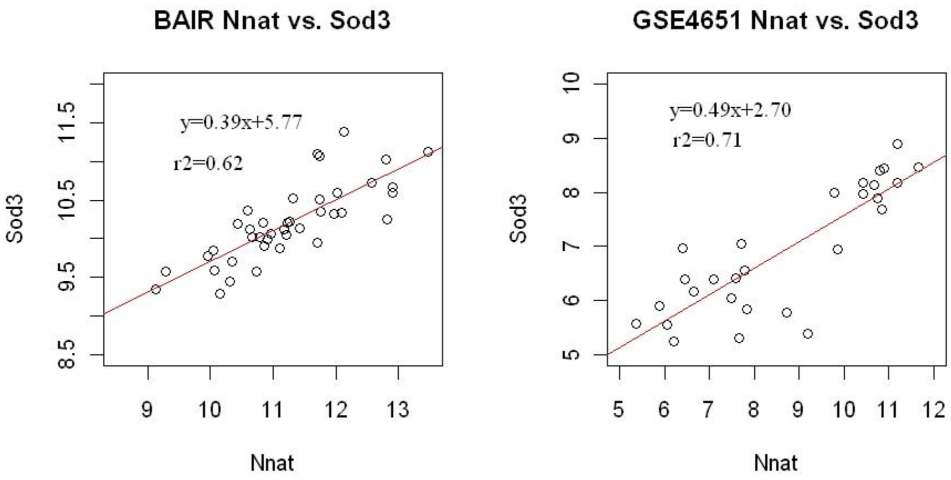

d. Expression of Nnat and Sod3

4. **Supplementary table 1**. Core enrichment genes of Nnat in common Kegg pathways between mouse WAT and hypothalamus

GENE	RANK.IN.GENE.LIST	RANK.METRIC.SCORE	RUNNING.ES	Data	Pathways
UQCR	6282	-0.18	-0.62	BAIR.WAT	OXIDATIVE_PHOSPHORYLATION
COX8C	6304	-0.18	-0.62	BAIR.WAT	OXIDATIVE_PHOSPHORYLATION
NDUFA4	6318	-0.18	-0.61	BAIR.WAT	OXIDATIVE_PHOSPHORYLATION
SDHA	6397	-0.19	-0.62	BAIR.WAT	OXIDATIVE_PHOSPHORYLATION
NDUFS7	6502	-0.2	-0.62	BAIR.WAT	OXIDATIVE_PHOSPHORYLATION
ATP6V0C	6533	-0.2	-0.62	BAIR.WAT	OXIDATIVE_PHOSPHORYLATION
ATP6V1A	6546	-0.21	-0.61	BAIR.WAT	OXIDATIVE_PHOSPHORYLATION
ATP5J2	6712	-0.22	-0.62	BAIR.WAT	OXIDATIVE_PHOSPHORYLATION
ATP6V1F	6730	-0.22	-0.61	BAIR.WAT	OXIDATIVE_PHOSPHORYLATION
NDUFS1	6737	-0.22	-0.61	BAIR.WAT	OXIDATIVE_PHOSPHORYLATION
NDUFB4	6805	-0.23	-0.61	BAIR.WAT	OXIDATIVE_PHOSPHORYLATION
COX4I1	6825	-0.23	-0.6	BAIR.WAT	OXIDATIVE_PHOSPHORYLATION
NDUFS6	6848	-0.23	-0.59	BAIR.WAT	OXIDATIVE_PHOSPHORYLATION
COX6B1	6946	-0.24	-0.6	BAIR.WAT	OXIDATIVE_PHOSPHORYLATION
ATP5G3	6989	-0.25	-0.59	BAIR.WAT	OXIDATIVE_PHOSPHORYLATION
ATP6V0D2	7046	-0.25	-0.59	BAIR.WAT	OXIDATIVE_PHOSPHORYLATION
ATP5J	7074	-0.26	-0.58	BAIR.WAT	OXIDATIVE_PHOSPHORYLATION
COX15	7075	-0.26	-0.57	BAIR.WAT	OXIDATIVE_PHOSPHORYLATION
NDUFV1	7084	-0.26	-0.56	BAIR.WAT	OXIDATIVE_PHOSPHORYLATION
ATP6V0A2	7086	-0.26	-0.56	BAIR.WAT	OXIDATIVE_PHOSPHORYLATION
NDUFAB1	7118	-0.26	-0.55	BAIR.WAT	OXIDATIVE_PHOSPHORYLATION

NDUFB9	7133	-0.26	-0.54	BAIR.WAT	OXIDATIVE_PHOSPHORYLATION
NDUFA9	7167	-0.27	-0.54	BAIR.WAT	OXIDATIVE_PHOSPHORYLATION
SDHC	7170	-0.27	-0.53	BAIR.WAT	OXIDATIVE_PHOSPHORYLATION
NDUFA10	7171	-0.27	-0.52	BAIR.WAT	OXIDATIVE_PHOSPHORYLATION
ATP6V1H	7178	-0.27	-0.51	BAIR.WAT	OXIDATIVE_PHOSPHORYLATION
COX5B	7325	-0.29	-0.51	BAIR.WAT	OXIDATIVE_PHOSPHORYLATION
ATP5D	7342	-0.29	-0.51	BAIR.WAT	OXIDATIVE_PHOSPHORYLATION
UQCRFS1	7353	-0.29	-0.5	BAIR.WAT	OXIDATIVE_PHOSPHORYLATION
NDUFS3	7369	-0.29	-0.49	BAIR.WAT	OXIDATIVE_PHOSPHORYLATION
CYC1	7382	-0.29	-0.48	BAIR.WAT	OXIDATIVE_PHOSPHORYLATION
NDUFC1	7389	-0.29	-0.47	BAIR.WAT	OXIDATIVE_PHOSPHORYLATION
NDUFB11	7475	-0.3	-0.47	BAIR.WAT	OXIDATIVE_PHOSPHORYLATION
NDUFA12	7495	-0.3	-0.46	BAIR.WAT	OXIDATIVE_PHOSPHORYLATION
SDHB	7519	-0.31	-0.45	BAIR.WAT	OXIDATIVE_PHOSPHORYLATION
NDUFB10	7558	-0.31	-0.44	BAIR.WAT	OXIDATIVE_PHOSPHORYLATION
NDUFS2	7564	-0.31	-0.43	BAIR.WAT	OXIDATIVE_PHOSPHORYLATION
NDUFS8	7602	-0.32	-0.43	BAIR.WAT	OXIDATIVE_PHOSPHORYLATION
ATP5G2	7631	-0.32	-0.42	BAIR.WAT	OXIDATIVE_PHOSPHORYLATION
ATP5E	7670	-0.32	-0.41	BAIR.WAT	OXIDATIVE_PHOSPHORYLATION
ATP6V1C1	7712	-0.33	-0.4	BAIR.WAT	OXIDATIVE_PHOSPHORYLATION
COX6C	7732	-0.33	-0.39	BAIR.WAT	OXIDATIVE_PHOSPHORYLATION
UQCRC1	7736	-0.33	-0.38	BAIR.WAT	OXIDATIVE_PHOSPHORYLATION
COX7C	7808	-0.34	-0.38	BAIR.WAT	OXIDATIVE_PHOSPHORYLATION
NDUFA2	7822	-0.34	-0.37	BAIR.WAT	OXIDATIVE_PHOSPHORYLATION
ATP5F1	7958	-0.36	-0.37	BAIR.WAT	OXIDATIVE_PHOSPHORYLATION
UQCRC2	7959	-0.36	-0.36	BAIR.WAT	OXIDATIVE_PHOSPHORYLATION
ATP5H	7960	-0.36	-0.34	BAIR.WAT	OXIDATIVE_PHOSPHORYLATION
ATP5A1	7986	-0.36	-0.33	BAIR.WAT	OXIDATIVE_PHOSPHORYLATION
ATP5O	8007	-0.36	-0.32	BAIR.WAT	OXIDATIVE_PHOSPHORYLATION
NDUFB2	8182	-0.39	-0.33	BAIR.WAT	OXIDATIVE_PHOSPHORYLATION
ATP5C1	8196	-0.39	-0.32	BAIR.WAT	OXIDATIVE_PHOSPHORYLATION
UQCRQ	8230	-0.4	-0.31	BAIR.WAT	OXIDATIVE_PHOSPHORYLATION
NDUFB5	8231	-0.4	-0.29	BAIR.WAT	OXIDATIVE_PHOSPHORYLATION
NDUFA5	8300	-0.41	-0.28	BAIR.WAT	OXIDATIVE_PHOSPHORYLATION
NDUFB6	8364	-0.43	-0.28	BAIR.WAT	OXIDATIVE_PHOSPHORYLATION
PPA1	8507	-0.45	-0.28	BAIR.WAT	OXIDATIVE_PHOSPHORYLATION
NDUFB7	8520	-0.46	-0.26	BAIR.WAT	OXIDATIVE_PHOSPHORYLATION
NDUFA8	8598	-0.47	-0.25	BAIR.WAT	OXIDATIVE_PHOSPHORYLATION
ATP6V1B2	8629	-0.48	-0.24	BAIR.WAT	OXIDATIVE_PHOSPHORYLATION
ATP5L	8630	-0.48	-0.22	BAIR.WAT	OXIDATIVE_PHOSPHORYLATION
ATP6V0B	8721	-0.5	-0.21	BAIR.WAT	OXIDATIVE_PHOSPHORYLATION
NDUFS4	8722	-0.5	-0.2	BAIR.WAT	OXIDATIVE_PHOSPHORYLATION
SDHD	8725	-0.5	-0.18	BAIR.WAT	OXIDATIVE_PHOSPHORYLATION
COX7A2	8769	-0.51	-0.16	BAIR.WAT	OXIDATIVE_PHOSPHORYLATION
ATP6V1D	8857	-0.54	-0.16	BAIR.WAT	OXIDATIVE_PHOSPHORYLATION
ATP5G1	8874	-0.54	-0.14	BAIR.WAT	OXIDATIVE_PHOSPHORYLATION

NDUFA11	8966	-0.57	-0.13	BAIR.WAT	OXIDATIVE_PHOSPHORYLATION
ATP6V1G1	8973	-0.57	-0.11	BAIR.WAT	OXIDATIVE_PHOSPHORYLATION
COX5A	8979	-0.57	-0.09	BAIR.WAT	OXIDATIVE_PHOSPHORYLATION
ATP6V1E1	9048	-0.61	-0.07	BAIR.WAT	OXIDATIVE_PHOSPHORYLATION
NDUFV2	9054	-0.61	-0.05	BAIR.WAT	OXIDATIVE_PHOSPHORYLATION
NDUFA6	9128	-0.65	-0.04	BAIR.WAT	OXIDATIVE_PHOSPHORYLATION
UQCRH	9140	-0.66	-0.01	BAIR.WAT	OXIDATIVE_PHOSPHORYLATION
ATP6V0D1	9149	-0.67	0.01	BAIR.WAT	OXIDATIVE_PHOSPHORYLATION
RPS28	5902	-0.1354583	-0.5792885	BAIR.WAT	RIBOSOME
RPS10	5904	-0.1356569	-0.570814	BAIR.WAT	RIBOSOME
RPL21	5947	-0.1397559	-0.5665496	BAIR.WAT	RIBOSOME
RPL22L1	6105	-0.1551711	-0.5738467	BAIR.WAT	RIBOSOME
RPL13	6164	-0.1622925	-0.5699006	BAIR.WAT	RIBOSOME
RPL35A	6196	-0.1659935	-0.5627769	BAIR.WAT	RIBOSOME
RPS9	6377	-0.1857755	-0.5706449	BAIR.WAT	RIBOSOME
RPS23	6391	-0.1880518	-0.5601632	BAIR.WAT	RIBOSOME
RPS16	6459	-0.1940979	-0.5551858	BAIR.WAT	RIBOSOME
RPL37	6544	-0.2046794	-0.5513921	BAIR.WAT	RIBOSOME
RPL13A	6726	-0.2221756	-0.5570658	BAIR.WAT	RIBOSOME
RPS24	6923	-0.2409211	-0.5631887	BAIR.WAT	RIBOSOME
RPS18	7072	-0.2565746	-0.5630884	BAIR.WAT	RIBOSOME
RPL36A	7103	-0.2595938	-0.5499332	BAIR.WAT	RIBOSOME
RPL35	7416	-0.2962655	-0.5652	BAIR.WAT	RIBOSOME
RPS7	7432	-0.2981387	-0.5479707	BAIR.WAT	RIBOSOME
RPL14	7535	-0.3081443	-0.5395926	BAIR.WAT	RIBOSOME
RPL8	7641	-0.3190589	-0.5308509	BAIR.WAT	RIBOSOME
RPS26	7826	-0.3409964	-0.5293334	BAIR.WAT	RIBOSOME
RPL19	7917	-0.3514223	-0.5169087	BAIR.WAT	RIBOSOME
RPL6	7938	-0.3532439	-0.4967377	BAIR.WAT	RIBOSOME
RPL10A	7954	-0.3551149	-0.4759032	BAIR.WAT	RIBOSOME
RPS25	7969	-0.3573998	-0.4548151	BAIR.WAT	RIBOSOME
RPS8	7970	-0.3574429	-0.4321981	BAIR.WAT	RIBOSOME
RPL26	8019	-0.3636389	-0.4144217	BAIR.WAT	RIBOSOME
RPL32	8020	-0.3636536	-0.3914117	BAIR.WAT	RIBOSOME
FAU	8149	-0.3825974	-0.381157	BAIR.WAT	RIBOSOME
RPL27A	8224	-0.395438	-0.364203	BAIR.WAT	RIBOSOME
RPL31	8282	-0.4084618	-0.3445717	BAIR.WAT	RIBOSOME
RPL12	8372	-0.4304691	-0.3270364	BAIR.WAT	RIBOSOME
MRPS7	8414	-0.4378241	-0.3038029	BAIR.WAT	RIBOSOME
RPS15A	8545	-0.4617216	-0.2887597	BAIR.WAT	RIBOSOME
RPL11	8606	-0.4731683	-0.2653611	BAIR.WAT	RIBOSOME
RPL18A	8620	-0.4755684	-0.236687	BAIR.WAT	RIBOSOME
RPL41	8622	-0.4759323	-0.2066816	BAIR.WAT	RIBOSOME
RPS2	8678	-0.4876633	-0.1818208	BAIR.WAT	RIBOSOME
RPL36AL	8709	-0.494244	-0.1538182	BAIR.WAT	RIBOSOME
RPS3	8732	-0.4995591	-0.1246072	BAIR.WAT	RIBOSOME

RPL7	8761	-0.5058685	-0.095651	BAIR.WAT	RIBOSOME
RPS5	8846	-0.5348876	-0.0709636	BAIR.WAT	RIBOSOME
RPL18	8914	-0.5537412	-0.0432299	BAIR.WAT	RIBOSOME
RPSA	8987	-0.5774723	-0.0145397	BAIR.WAT	RIBOSOME
RPL37A	9104	-0.6363922	0.01308188	BAIR.WAT	RIBOSOME
SEC61B	6325	-0.1810245	-0.5200132	BAIR.WAT	CHOLERA_INFECTION
SEC61G	6424	-0.1907431	-0.5130916	BAIR.WAT	CHOLERA_INFECTION
ATP6V0C	6533	-0.2031062	-0.5061182	BAIR.WAT	CHOLERA_INFECTION
ATP6V1A	6546	-0.2054349	-0.4884864	BAIR.WAT	CHOLERA_INFECTION
ACTG1	6579	-0.2085971	-0.4727389	BAIR.WAT	CHOLERA_INFECTION
ATP6V1F	6730	-0.2225913	-0.4685386	BAIR.WAT	CHOLERA_INFECTION
SEC61A2	6916	-0.2404175	-0.4665027	BAIR.WAT	CHOLERA_INFECTION
ATP6V0D2	7046	-0.2535169	-0.457167	BAIR.WAT	CHOLERA_INFECTION
ATP6V0A2	7086	-0.258147	-0.4376134	BAIR.WAT	CHOLERA_INFECTION
ATP6V1H	7178	-0.2678407	-0.4228233	BAIR.WAT	CHOLERA_INFECTION
ARF3	7488	-0.3038829	-0.4284271	BAIR.WAT	CHOLERA_INFECTION
ATP6V1C1	7712	-0.3272635	-0.4225197	BAIR.WAT	CHOLERA_INFECTION
ADCY9	7810	-0.338717	-0.4018488	BAIR.WAT	CHOLERA_INFECTION
ERO1L	7992	-0.3600593	-0.388349	BAIR.WAT	CHOLERA_INFECTION
ARF5	8006	-0.3619993	-0.3563936	BAIR.WAT	CHOLERA_INFECTION
PDIA4	8519	-0.4564703	-0.370016	BAIR.WAT	CHOLERA_INFECTION
ARF6	8526	-0.457307	-0.3285134	BAIR.WAT	CHOLERA_INFECTION
ADCY3	8579	-0.4683415	-0.290998	BAIR.WAT	CHOLERA_INFECTION
ATP6V0B	8721	-0.4977501	-0.260454	BAIR.WAT	CHOLERA_INFECTION
ARF1	8741	-0.5014337	-0.216298	BAIR.WAT	CHOLERA_INFECTION
SEC61A1	8818	-0.5240427	-0.176259	BAIR.WAT	CHOLERA_INFECTION
ATP6V1D	8857	-0.539169	-0.1306915	BAIR.WAT	CHOLERA_INFECTION
ATP6V1G1	8973	-0.5716066	-0.0905108	BAIR.WAT	CHOLERA_INFECTION
ATP6V1E1	9048	-0.608306	-0.0424866	BAIR.WAT	CHOLERA_INFECTION
ATP6V0D1	9149	-0.6674306	0.00815923	BAIR.WAT	CHOLERA_INFECTION
ATP6V0D1	48	0.75425029	0.06066386	GSEA6514.Hypo	CHOLERA_INFECTION
ARF5	104	0.71975589	0.11767986	GSEA6514.Hypo	CHOLERA_INFECTION
SEC61A2	256	0.67447311	0.16166064	GSEA6514.Hypo	CHOLERA_INFECTION
AK1	305	0.6586256	0.21405539	GSEA6514.Hypo	CHOLERA_INFECTION
ATP6V0C	750	0.57445556	0.221554	GSEA6514.Hypo	CHOLERA_INFECTION
ATP6V1G2	751	0.57393199	0.2711846	GSEA6514.Hypo	CHOLERA_INFECTION
ATP6V0A1	971	0.54513216	0.29752105	GSEA6514.Hypo	CHOLERA_INFECTION
ARF3	1051	0.5351693	0.33629516	GSEA6514.Hypo	CHOLERA_INFECTION
ATP6V0B	1130	0.52556008	0.3743333	GSEA6514.Hypo	CHOLERA_INFECTION
ATP6V1C1	1168	0.5221566	0.41597185	GSEA6514.Hypo	CHOLERA_INFECTION
ACTG1	1368	0.49841017	0.44016793	GSEA6514.Hypo	CHOLERA_INFECTION
ARF1	1463	0.48905423	0.47352934	GSEA6514.Hypo	CHOLERA_INFECTION
ATP6V1E1	1703	0.46028858	0.4906291	GSEA6514.Hypo	CHOLERA_INFECTION
PDIA4	1943	0.43223706	0.50530314	GSEA6514.Hypo	CHOLERA_INFECTION
ATP6V1A	2282	0.39292249	0.5071731	GSEA6514.Hypo	CHOLERA_INFECTION
ATP6V1F	2397	0.38072288	0.5292667	GSEA6514.Hypo	CHOLERA_INFECTION

ARF4	2554	0.36443222	0.5459618	GSEA6514.Hypo	CHOLERA_INFECTION
RPL21	7980	-0.215069	-0.4390552	GSEA6514.Hypo	RIBOSOME
RPS7	8011	-0.2185133	-0.4283264	GSEA6514.Hypo	RIBOSOME
RPL7	8087	-0.2258599	-0.4214238	GSEA6514.Hypo	RIBOSOME
RPL41	8186	-0.2388746	-0.4159011	GSEA6514.Hypo	RIBOSOME
RPL10A	8408	-0.2624991	-0.4206162	GSEA6514.Hypo	RIBOSOME
RPL11	8522	-0.2746218	-0.4142989	GSEA6514.Hypo	RIBOSOME
RPL31	8575	-0.2835957	-0.401618	GSEA6514.Hypo	RIBOSOME
FAU	8725	-0.3030172	-0.3969618	GSEA6514.Hypo	RIBOSOME
RPS28	8933	-0.3269751	-0.3963363	GSEA6514.Hypo	RIBOSOME
RPL37	8967	-0.3314468	-0.3788724	GSEA6514.Hypo	RIBOSOME
RPL37A	9045	-0.3409406	-0.3650061	GSEA6514.Hypo	RIBOSOME
RPL28	9058	-0.3427393	-0.3448415	GSEA6514.Hypo	RIBOSOME
RPS21	9194	-0.3592721	-0.3353557	GSEA6514.Hypo	RIBOSOME
RPS25	9241	-0.3660164	-0.31698	GSEA6514.Hypo	RIBOSOME
RPS20	9323	-0.3761964	-0.3013027	GSEA6514.Hypo	RIBOSOME
RPS15A	9394	-0.3844281	-0.2840667	GSEA6514.Hypo	RIBOSOME
RPL35	9415	-0.3861425	-0.2619653	GSEA6514.Hypo	RIBOSOME
RPS23	9516	-0.3986951	-0.2466976	GSEA6514.Hypo	RIBOSOME
RPL26	9529	-0.4001468	-0.2229643	GSEA6514.Hypo	RIBOSOME
RPL32	9560	-0.4038346	-0.2007149	GSEA6514.Hypo	RIBOSOME
RPS16	9574	-0.4052853	-0.1767573	GSEA6514.Hypo	RIBOSOME
RPL23A	9626	-0.4107626	-0.1560758	GSEA6514.Hypo	RIBOSOME
RPL36A	9655	-0.4140722	-0.1329996	GSEA6514.Hypo	RIBOSOME
RPS24	9673	-0.4178868	-0.1086394	GSEA6514.Hypo	RIBOSOME
MRPL13	9681	-0.4186401	-0.0832805	GSEA6514.Hypo	RIBOSOME
RPL22L1	9708	-0.4219686	-0.0595231	GSEA6514.Hypo	RIBOSOME
RPL35A	9782	-0.4319815	-0.0396164	GSEA6514.Hypo	RIBOSOME
RPS27	9860	-0.4431706	-0.0193948	GSEA6514.Hypo	RIBOSOME
RPS11	9877	-0.444847	0.00673665	GSEA6514.Hypo	RIBOSOME
RPL36AL	9994	-0.4632901	0.02449715	GSEA6514.Hypo	RIBOSOME
RPL39	10026	-0.4678826	0.050633	GSEA6514.Hypo	RIBOSOME
NDUFA6	8884	-0.3220876	-0.3396046	GSEA6514.Hypo	OXIDATIVE_PHOSPHORYLATION
NDUFA11	8980	-0.3334448	-0.338466	GSEA6514.Hypo	OXIDATIVE_PHOSPHORYLATION
NDUFB5	8995	-0.3348385	-0.3295453	GSEA6514.Hypo	OXIDATIVE_PHOSPHORYLATION
ATP5G1	9047	-0.3410569	-0.3239693	GSEA6514.Hypo	OXIDATIVE_PHOSPHORYLATION
NDUFB7	9113	-0.3496626	-0.3194674	GSEA6514.Hypo	OXIDATIVE_PHOSPHORYLATION
UQCR	9175	-0.3565984	-0.3143708	GSEA6514.Hypo	OXIDATIVE_PHOSPHORYLATION
NDUFC1	9226	-0.3641129	-0.3079929	GSEA6514.Hypo	OXIDATIVE_PHOSPHORYLATION
NDUFB6	9269	-0.3688861	-0.3007044	GSEA6514.Hypo	OXIDATIVE_PHOSPHORYLATION
UQCRC2	9286	-0.3710566	-0.2908652	GSEA6514.Hypo	OXIDATIVE_PHOSPHORYLATION
ATP5H	9338	-0.3775582	-0.284171	GSEA6514.Hypo	OXIDATIVE_PHOSPHORYLATION
ATP5J2	9393	-0.3842707	-0.2775578	GSEA6514.Hypo	OXIDATIVE_PHOSPHORYLATION
NDUFB3	9403	-0.3851249	-0.2666187	GSEA6514.Hypo	OXIDATIVE_PHOSPHORYLATION
NDUFS4	9454	-0.3906234	-0.2594287	GSEA6514.Hypo	OXIDATIVE_PHOSPHORYLATION
NDUFA13	9460	-0.3912874	-0.2479186	GSEA6514.Hypo	OXIDATIVE_PHOSPHORYLATION

ATP5O	9503	-0.3971408	-0.2397645	GSEA6514.Hypo	OXIDATIVE_PHOSPHORYLATION
ATP5E	9507	-0.3979985	-0.2278578	GSEA6514.Hypo	OXIDATIVE_PHOSPHORYLATION
NDUFA12	9589	-0.4067827	-0.2231346	GSEA6514.Hypo	OXIDATIVE_PHOSPHORYLATION
ATP5L	9631	-0.4111172	-0.2144568	GSEA6514.Hypo	OXIDATIVE_PHOSPHORYLATION
NDUFB9	9667	-0.4163418	-0.2050456	GSEA6514.Hypo	OXIDATIVE_PHOSPHORYLATION
NDUFA7	9688	-0.4195801	-0.194102	GSEA6514.Hypo	OXIDATIVE_PHOSPHORYLATION
COX6B1	9748	-0.4261748	-0.1866827	GSEA6514.Hypo	OXIDATIVE_PHOSPHORYLATION
ATP5I	9762	-0.4292582	-0.1747737	GSEA6514.Hypo	OXIDATIVE_PHOSPHORYLATION
NDUFB4	9848	-0.4415597	-0.1693673	GSEA6514.Hypo	OXIDATIVE_PHOSPHORYLATION
PPA2	9854	-0.4421825	-0.156298	GSEA6514.Hypo	OXIDATIVE_PHOSPHORYLATION
COX6C	9893	-0.4465218	-0.1462489	GSEA6514.Hypo	OXIDATIVE_PHOSPHORYLATION
UQCRH	9898	-0.4475095	-0.1329208	GSEA6514.Hypo	OXIDATIVE_PHOSPHORYLATION
NDUFA2	9959	-0.4579307	-0.1246242	GSEA6514.Hypo	OXIDATIVE_PHOSPHORYLATION
NDUFS6	9995	-0.463303	-0.1137742	GSEA6514.Hypo	OXIDATIVE_PHOSPHORYLATION
NDUFA5	9996	-0.4635981	-0.0995711	GSEA6514.Hypo	OXIDATIVE_PHOSPHORYLATION
NDUFA3	10024	-0.4675551	-0.0878265	GSEA6514.Hypo	OXIDATIVE_PHOSPHORYLATION
COX17	10050	-0.4711683	-0.0757801	GSEA6514.Hypo	OXIDATIVE_PHOSPHORYLATION
NDUFB8	10054	-0.4722995	-0.0615971	GSEA6514.Hypo	OXIDATIVE_PHOSPHORYLATION
ATP5J	10068	-0.4745021	-0.048302	GSEA6514.Hypo	OXIDATIVE_PHOSPHORYLATION
NDUFB11	10078	-0.4765361	-0.0345624	GSEA6514.Hypo	OXIDATIVE_PHOSPHORYLATION
NDUFA1	10181	-0.4961127	-0.029109	GSEA6514.Hypo	OXIDATIVE_PHOSPHORYLATION
NDUFA4	10218	-0.5011539	-0.0171949	GSEA6514.Hypo	OXIDATIVE_PHOSPHORYLATION
COX7A2	10285	-0.5153352	-0.0077129	GSEA6514.Hypo	OXIDATIVE_PHOSPHORYLATION
COX6A2	10351	-0.5328622	0.00240168	GSEA6514.Hypo	OXIDATIVE_PHOSPHORYLATION
UQCRB	10441	-0.5640648	0.01117906	GSEA6514.Hypo	OXIDATIVE_PHOSPHORYLATION

5. **Supplementary table 2**. Canonical pathways (p-value < 0.05 & FDR < 0.25) of *Nnat* in BAIR fat-feeding mice.

NAME	SIZE	ES	NES	p.val	FDR
HSA00190_OXIDATIVE_PHOSPHORYLATION	92	-0.63	-2.99	0	0
HSA03050_PROTEASOME	22	-0.83	-2.79	0	0
OXIDATIVE_PHOSPHORYLATION	48	-0.64	-2.63	0	0
PROTEASOMEPATHWAY	21	-0.77	-2.55	0	0
HSA03010_RIBOSOME	52	-0.59	-2.52	0	0
FLAGELLAR_ASSEMBLY	15	-0.67	-2.07	0	0.0037
TYPE_III_SECRETION_SYSTEM	15	-0.67	-2.06	0.002	0.0037
HSA05110_CHOLERA_INFECTION	33	-0.54	-2.06	0	0.0033
ATP_SYNTHESIS	15	-0.67	-2.04	0	0.0033
PHOTOSYNTHESIS	15	-0.67	-2.04	0	0.0030
RIBOSOMAL_PROTEINS	64	-0.46	-2.04	0	0.0027
PROTEASOME	16	-0.65	-2.00	0	0.0044
TRANSLATION_FACTORS	40	-0.50	-1.93	0	0.0081
GLUTAMATE_METABOLISM	15	-0.60	-1.84	0.0044	0.0202
HSA00450_SELENOAMINO_ACID_METABOLISM	19	-0.55	-1.76	0.0021	0.0367
HSA03022_BASAL_TRANSCRIPTION_FACTORS	21	-0.50	-1.68	0.0194	0.0689
KREBS_TCA_CYCLE	26	-0.46	-1.65	0.0164	0.0842
HSA00510_N_GLYCAN_BIOSYNTHESIS	28	-0.46	-1.64	0.0105	0.0846
UBIQUITIN_MEDIATED_PROTEOLYSIS	19	-0.50	-1.61	0.0157	0.0967
HSA00252_ALANINE_AND_ASPARTATE_METABOLISM	21	-0.48	-1.61	0.0294	0.0946

PYRUVATE_METABOLISM	27	-0.43	-1.58	0.0188	0.1116
HSA00251_GLUTAMATE_METABOLISM	20	-0.47	-1.56	0.0351	0.1191
MRNA_PROCESSING_REACTOME	98	-0.33	-1.56	0.0022	0.1168
HSA00330_ARGININE_AND_PROLINE_METABOLISM	15	-0.51	-1.55	0.0298	0.1181
RNA_TRANSCRIPTION_REACTOME	31	-0.41	-1.53	0.0394	0.1328
GLYCOLYSIS_AND_GLUCONEOGENESIS	25	-0.43	-1.51	0.0462	0.1461
PYRIMIDINE_METABOLISM	40	-0.38	-1.50	0.0300	0.1487
HSA00240_PYRIMIDINE_METABOLISM	58	-0.34	-1.45	0.0359	0.1935
PURINE_METABOLISM	80	-0.31	-1.43	0.0215	0.1994
HSA01430_CELL_COMMUNICATION	32	0.67	2.46	0	0
HSA04060_CYTOKINE_CYTOKINE_RECEPTOR_INTERACTION	62	0.55	2.38	0	0
HSA04512_ECM_RECEPTOR_INTERACTION	44	0.58	2.31	0	0
HSA04510_FOCAL_ADHESION	127	0.47	2.25	0	0.0006
INTEGRIN_MEDIATED_CELL_ADHESION_KEGG	54	0.52	2.18	0	0.0007
HSA04610_COMPLEMENT_AND_COAGULATION_CASCADES	24	0.61	2.06	0	0.0029
HSA04080_NEUROACTIVE_LIGAND_RECEPTOR_INTERACTION	36	0.52	1.97	0	0.0109
HSA04670_LEUKOCYTE_TRANSENDOTHELIAL_MIGRATION	66	0.45	1.93	0	0.0179
GPCRDB_CLASS_A_RHODOPSIN_LIKE	21	0.57	1.83	0.0037	0.0511
SA_B_CELL_RECEPTOR_COMPLEXES	17	0.58	1.75	0.0118	0.0973
HSA04630_JAK_STAT_SIGNALING_PATHWAY	65	0.41	1.75	0	0.0914
INTEGRINPATHWAY	30	0.47	1.70	0.0110	0.1253
HSA04514_CELL_ADHESION_MOLECULES	39	0.44	1.69	0.0073	0.1257
HSA04360_AXON_GUIDANCE	69	0.39	1.69	0.0017	0.1172
HSA04350_TGF_BETA_SIGNALING_PATHWAY	53	0.41	1.68	0.0052	0.1213
BREAST_CANCER_ESTROGEN_SIGNALING	45	0.43	1.67	0.0092	0.1178
HSA04916_MELANOGENESIS	52	0.41	1.64	0.0037	0.1388
PROSTAGLANDIN_SYNTHESIS_REGULATION	19	0.50	1.64	0.0214	0.1370
ST_INTEGRIN_SIGNALING_PATHWAY	55	0.38	1.56	0.0120	0.2298
VIPPATHWAY	18	0.48	1.55	0.0287	0.2379

6. **Supplementary table 3**. Canonical pathways (p-value < 0.05 & FDR < 0.25) of *NNAT* in human fat tissue.

NAME	SIZE	ES	NES	p.val	FDR
HSA03010_RIBOSOME	52	0.77	3.11	0	0
RIBOSOMAL_PROTEINS	73	0.72	3.10	0	0
TRANSLATION_FACTORS	38	0.60	2.26	0	0
DNA_REPLICATION_REACTOME	29	0.52	1.81	0.0016	0.0797
RNA_TRANSCRIPTION_REACTOME	28	0.49	1.70	0.0047	0.1939
HSA00071_FATTY_ACID_METABOLISM	33	0.46	1.67	0.0139	0.2199
HSA01030_GLYCAN_STRUCTURES_BIOSYNTHESIS_1	39	-0.61	-2.47	0	0
HSA04512_ECM_RECEPTOR_INTERACTION	50	-0.56	-2.46	0	0
HSA01430_CELL_COMMUNICATION	52	-0.56	-2.41	0	0
HSA04940_TYPE_I_DIABETES_MELLITUS	24	-0.65	-2.37	0	0
HSA04514_CELL_ADHESION_MOLECULES	59	-0.51	-2.28	0	0.0003
HSA04510_FOCAL_ADHESION	127	-0.44	-2.28	0	0.0002
HSA04610_COMPLEMENT_AND_COAGULATION_CASCADES	34	-0.56	-2.25	0	0.0013
SMOOTH_MUSCLE_CONTRACTION	89	-0.44	-2.16	0	0.0036
HSA04612_ANTIGEN_PROCESSING_AND_PRESENTATION	42	-0.49	-2.09	0	0.0070
HSA00510_N_GLYCAN_BIOSYNTHESIS	19	-0.59	-2.03	0.0027	0.0102
HSA00600_SPHINGOLIPID_METABOLISM	15	-0.63	-2.01	0.0024	0.0108
HSA04640_HEMATOPOIETIC_CELL_LINEAGE	37	-0.50	-2.01	0	0.0105
PROSTAGLANDIN_SYNTHESIS_REGULATION	17	-0.61	-1.98	0.0025	0.0134
IL1RPATHWAY	20	-0.57	-1.98	0	0.0126
HSA04060_CYTOKINE_CYTOKINE_RECEPTOR_INTERACTION	103	-0.39	-1.93	0	0.0164
CDMACPATHWAY	15	-0.60	-1.90	0.0026	0.0211

HSA04670_LEUKOCYTE_TRANSENDOTHELIAL_MIGRATION	57	-0.40	-1.79	0.0028	0.0502
HSA04620_TOLL_LIKE_RECEPTOR_SIGNALING_PATHWAY	62	-0.39	-1.78	0	0.0538
NFKBPATHWAY	17	-0.54	-1.77	0.0049	0.0519
GSK3PATHWAY	19	-0.52	-1.76	0.0150	0.0544
HSA05219_BLADDER_CANCER	32	-0.44	-1.72	0.0109	0.0691
HSA04650_NATURAL_KILLER_CELL_MEDIATED_CYTOTOXICITY	64	-0.38	-1.71	0.0030	0.0717
INFLAMPATHWAY	16	-0.53	-1.70	0.0298	0.0732
HSA05222_SMALL_CELL_LUNG_CANCER	60	-0.38	-1.69	0.0056	0.0738
HSA05130_PATHOGENIC_ESCHERICHIA_COLI_INFECTION_EHEC	25	-0.45	-1.67	0.0080	0.0802
HSA05218_MELANOMA	42	-0.39	-1.67	0.0057	0.0789
BREAST_CANCER_ESTROGEN_SIGNALING	67	-0.36	-1.65	0.0029	0.0838
HSA05131_PATHOGENIC_ESCHERICHIA_COLI_INFECTION_EPEC	25	-0.45	-1.65	0.0103	0.0818
CASPASEPATHWAY	15	-0.52	-1.65	0.0237	0.0801
HSA04662_B_CELL_RECEPTOR_SIGNALING_PATHWAY	36	-0.42	-1.65	0.0085	0.0786
HSA04010_MAPK_SIGNALING_PATHWAY	131	-0.32	-1.64	0	0.0827
HSA05120_EPITHELIAL_CELL_SIGNALING_IN_HELICOBACTER_PYLORI_INFECTION	46	-0.39	-1.63	0.0028	0.0845
HSA05210_COLORECTAL_CANCER	59	-0.36	-1.62	0.0055	0.0844
ST_TUMOR_NECROSIS_FACTOR_PATHWAY	19	-0.48	-1.61	0.0190	0.0888
HSA04630_JAK_STAT_SIGNALING_PATHWAY	75	-0.34	-1.59	0.0032	0.1018
APOPTOSIS_GENMAPP	34	-0.39	-1.59	0.0242	0.1007
PROTEASOMEPATHWAY	20	-0.47	-1.58	0.0267	0.0987
EDG1PATHWAY	21	-0.44	-1.55	0.0403	0.1219
HSA04810_REGULATION_OF_ACTIN_CYTOSKELETON	117	-0.30	-1.54	0.0034	0.1224
HSA01032_GLYCAN_STRUCTURES_DEGRADATION	16	-0.47	-1.54	0.0416	0.1200
SIG_REGULATION_OF_THE_ACTIN_CYTOSKELETON_BY_RHO_GTPASES	20	-0.46	-1.54	0.0339	0.1202
HSA04360_AXON_GUIDANCE	64	-0.33	-1.49	0.0245	0.1541
SA_B_CELL_RECEPTOR_COMPLEXES	17	-0.47	-1.49	0.0482	0.1567
HSA04540_GAP_JUNCTION	54	-0.32	-1.42	0.0351	0.2119

7. **Supplementary table 4**: Common canonical pathways associated with Nnat expression between fat-fed mice and human.

NAME	SIZE	ES	NES	p-val	FDR	Data
BREAST_CANCER_ESTROGEN_SIGNALING	45	0.43	1.67	0.009	0.118	BAIR
BREAST_CANCER_ESTROGEN_SIGNALING	67	-0.36	-1.65	0.003	0.084	Nair
HSA00510_N_GLYCAN_BIOSYNTHESIS	28	-0.46	-1.64	0.011	0.085	BAIR
HSA00510_N_GLYCAN_BIOSYNTHESIS	19	-0.59	-2.03	0.003	0.010	Nair
HSA01430_CELL_COMMUNICATION	32	0.67	2.46	0	0	BAIR
HSA01430_CELL_COMMUNICATION	52	-0.56	-2.41	0	0	Nair
HSA03010_RIBOSOME	52	-0.59	-2.52	0	0	BAIR
HSA03010_RIBOSOME	52	0.77	3.11	0	0	Nair
HSA04060_CYTOKINE_CYTOKINE_RECEPTOR_INTERACTION	62	0.55	2.38	0	0	BAIR
HSA04060_CYTOKINE_CYTOKINE_RECEPTOR_INTERACTION	103	-0.39	-1.93	0	0.016	Nair
HSA04360_AXON_GUIDANCE	69	0.39	1.69	0.002	0.117	BAIR
HSA04360_AXON_GUIDANCE	64	-0.33	-1.49	0.024	0.154	Nair
HSA04510_FOCAL_ADHESION	127	0.47	2.25	0	5.79E-04	BAIR
HSA04510_FOCAL_ADHESION	127	-0.44	-2.28	0	2.29E-04	Nair
HSA04512_ECM_RECEPTOR_INTERACTION	44	0.58	2.31	0	0	BAIR
HSA04512_ECM_RECEPTOR_INTERACTION	50	-0.56	-2.46	0	0	Nair
HSA04514_CELL_ADHESION_MOLECULES	39	0.44	1.69	0.007	0.126	BAIR
HSA04514_CELL_ADHESION_MOLECULES	59	-0.51	-2.28	0	2.75E-04	Nair

HSA04610_COMPLEMENT_AND_COAGULATION_CASCADES	24	0.61	2.06	0	0.003	BAIR
HSA04610_COMPLEMENT_AND_COAGULATION_CASCADES	34	-0.56	-2.25	0	0.001	Nair
HSA04630_JAK_STAT_SIGNALING_PATHWAY	65	0.41	1.75	0	0.091	BAIR
HSA04630_JAK_STAT_SIGNALING_PATHWAY	75	-0.34	-1.59	0.003	0.102	Nair
HSA04670_LEUKOCYTE_TRANSENDOTHELIAL_MIGRATION	66	0.45	1.93	0	0.018	BAIR
HSA04670_LEUKOCYTE_TRANSENDOTHELIAL_MIGRATION	57	-0.40	-1.79	0.003	0.050	Nair
HSA05130_PATHOGENIC_ESCHERICHIA_COLI_INFECTION_EHEC	25	-0.45	-1.67	0.008	0.080	Nair
HSA05131_PATHOGENIC_ESCHERICHIA_COLI_INFECTION_EPEC	25	-0.45	-1.65	0.010	0.082	Nair
PROSTAGLANDIN_SYNTHESIS_REGULATION	19	0.50	1.64	0.021	0.137	BAIR
PROSTAGLANDIN_SYNTHESIS_REGULATION	17	-0.61	-1.98	0.002	0.013	Nair
PROTEASOMEPATHWAY	21	-0.77	-2.55	0	0	BAIR
PROTEASOMEPATHWAY	20	-0.47	-1.58	0.027	0.099	Nair
RIBOSOMAL_PROTEINS	64	-0.46	-2.04	0	0.003	BAIR
RIBOSOMAL_PROTEINS	73	0.72	3.10	0	0	Nair
RNA_TRANSCRIPTION_REACTOME	31	-0.41	-1.53	0.039	0.133	BAIR
RNA_TRANSCRIPTION_REACTOME	28	0.49	1.70	0.005	0.194	Nair
SA_B_CELL_RECEPTOR_COMPLEXES	17	0.58	1.75	0.012	0.097	BAIR
SA_B_CELL_RECEPTOR_COMPLEXES	17	-0.47	-1.49	0.048	0.157	Nair
TRANSLATION_FACTORS	40	-0.50	-1.93	0	0.008	BAIR
TRANSLATION_FACTORS	38	0.60	2.26	0	0	Nair
